# Oligonucleotide Ligation Assay (OLA)-Simple: Field Implementation, Usability, and Performance of a near Point-of-Care HIV Drug Resistance Assay in Kenya

**DOI:** 10.3390/laboratories3010005

**Published:** 2026-02-04

**Authors:** Prestone O. Owiti, Bhavna H. Chohan, Ingrid A. Beck, Nuttada Panpradist, Pooja Maheria, Katherine K. Thomas, Jessica H. Giang, Leonard Kingwara, Vera M. Onwonga, Rukia S. Madada, Shalyn Akasa, Grace Akinyi, Valarie Opollo, John Kiiru, Nancy Bowen, Mansour Samadpour, Garoma W. Basha, Barry R. Lutz, Lisa M. Frenkel, Patrick Oyaro, Lisa L. Abuogi, Rena C. Patel

**Affiliations:** 1 Department of Paediatrics and Child Health, University of Nairobi, Nairobi P.O. Box 19676-00202, Kenya; 2 Department of Global Health, University of Washington, Seattle, WA 98195, USA; 3 Center for Global Infectious Disease Research, Seattle Children’s Research Institute, Seattle, WA 98109, USA; 4 Department of Bioengineering, University of Washington, Seattle, WA 98195, USA; 5 Department of Biomedical Engineering, The University of Texas at Austin, Austin, TX 78712, USA; 6 Department of Medicine, University of Alabama at Birmingham, Birmingham, AL 35294, USA; 7 School of Medicine, University of Washington, Seattle, WA 98195, USA; 8 National Public Health Institute, Nairobi P.O. Box 20750-00202, Kenya; 9 Center for Global Health Research (CGHR), Kenya Medical Research Institute, Kisumu P.O. Box 1578-40100, Kenya; 10 IEH Laboratories & Consulting Group, Seattle, WA 98125, USA; 11 Department of Medicine, University of Washington, Seattle, WA 98195, USA; 12 Department of Pediatrics, University of Washington, Seattle, WA 98195, USA; 13 Department of Laboratory Medicine and Pathology, University of Washington, Seattle, WA 98195, USA; 14 Health Innovations Kenya (HIK), Kisumu P.O. Box 2871-40100, Kenya; 15 Liverpool Voluntary Counselling and Testing (LVCT) Care and Treatment Health, Nairobi P.O. Box 19835-00202, Kenya; 16 Department of Pediatrics, University of Colorado, Denver, CO 80045, USA

**Keywords:** HIV drug mutations, resistance testing, near point-of-care, user-friendly, LMIC

## Abstract

A point-of-care (POC) HIV drug resistance (HIV-DR) test is needed for low- and middle-income countries (LMICs). Oligonucleotide Ligation Assay (OLA)-Simple, designed as a near-POC HIV-DR test, was assessed for its overall usability in Kenya by technicians with and without molecular laboratory PCR experience. Further, its diagnostic accuracy was evaluated by PCR-experienced technicians utilizing 147 plasma samples with known Sanger sequence genotypes—based on seven major HIV-DR mutations of nucleotide and non-nucleoside reverse transcriptase inhibitors. Thirteen laboratory technicians were recruited, including five with prior PCR experience. Twelve technicians completed the training and attained OLA-Simple testing competency, ten of whom were able to perform the OLA-Simple test within 6 h. Technicians’ survey feedback indicated the user-friendliness of OLA-Simple, citing straightforward reagent reconstitution, concise instructions in prompts, and a shorter sample-to-result test time compared to standard genotyping assays. Of the 147 archived plasma samples tested, 132 (90%) yielded interpretable results. OLA-Simple assay demonstrated a sensitivity of 97.3% (95% CI 94.5, 98.9), a specificity of 97.2% (95% CI 95.5, 98.3), and a percent agreement of 97.1% (95% CI 95.9, 98.2) compared to Sanger sequencing. This evaluation found that OLA-Simple was user-friendly among intended end-users and performed well. LMIC HIV programs would benefit from strategizing on case-use scenarios for such near-POC HIV-DR assays to improve HIV outcomes.

## 1. Introduction

Human immunodeficiency virus (HIV) drug resistance (HIV-DR) is an increasing threat to HIV treatment success and epidemic control globally [1]. In resource-rich countries, HIV-DR testing is used to guide initial antiretroviral therapy (ART) and subsequent modifications, whereas in low- and middle-income countries (LMICs), HIV-DR testing is limited to people living with HIV (PLWH) who repeatedly have high viral loads, despite ART and adherence optimization, and possibly only when on second line ART or salvage regimens [2]. There is growing momentum to increase the availability of HIV-DR testing in LMICs, and to assess HIV-DR in ART failure for patient-level decision-making [3,4].

As many as half a million PLWH in LMICs are estimated to require HIV-DR tests every year [5], but current HIV-DR testing falls far short of this need. Currently, HIV-DR testing is commonly performed by Sanger sequencing, which is costly to run, requires complex laboratory infrastructure, and has long turnaround times [6]. These barriers, together with other logistical challenges, such as the requirement for sustained cold chains during the transportation of test samples to centralized laboratories, and centralized clinical decision-making, make HIV-DR testing unavailable for routine patient care in LMICs [5,6]. As of early 2022, during this study, only 19 laboratories in sub-Saharan Africa had the capacity to sequence HIV-DR mutations (HIV-DRMs), of which only 11 were World Health Organization (WHO)-certified [7]. To scale up HIV-DR testing in LMICs, there is a dire need for new technologies that are potentially simpler, low-cost, and rapidly operable at or near the point-of-care (POC) [6].

POC HIV-DR tests under development in the past decade are designed to detect a few HIV-DR mutations of great clinical significance. Potentially, these tests could decentralize testing, reduce cost, and shorten the turn-around time [6,8]. One such near-POC HIV-DR test is based on an oligonucleotide ligation assay (OLA), whose performance is premised on the annealing of assay-specific probes at relatively low temperatures to accommodate viral polymorphisms, and the specificity of the ligase enzyme for the two bases on each side of ligation, enabling sensitive and specific detection of HIV-DR point mutations via a step-by-step, prompt-guided implementation [9,10]. The original OLA originally relied on a relatively laborious assay involving PCR, a ligation reaction, and an enzyme-linked immunosorbent assay (ELISA) procedure to detect the amplified and ligated DNA targets in samples of interest. However, simplifications and modifications to multiple steps have produced OLA-Simple, which detects HIV-DRMs as band lines on paper format lateral flow strips [11]. The test’s key reagents are lyophilized to make them compact and stable at a temperature of ~4 °C (in a refrigerator). The test’s procedures are executed through step-by-step user-in-the-loop guides that make complex assay methods implementable with relative ease [9,10]. Already, OLA-Simple’s performance assessment at the developer’s U.S.-based laboratory has demonstrated a high overall sensitivity and specificity on HIV samples of various subtypes, sourced from different countries [11,12]. While the test developer’s experienced laboratory staff report OLA-Simple’s simplicity [10,13], it is necessary to assess usability by the intended users in LMICs, like Kenya, which has an estimated 1.3 million adults and 75,000 children (aged 0 to 14 years) living with HIV. Over 98% of adults and 83% of children are on antiretroviral treatment (ART), necessitating regular virological monitoring. A subset of patients failing treatment (e.g., those on dolutegravir (DTG)-containing or second-line ART regimens) are usually recommended for HIV-DR testing [14].

To determine if the OLA-Simple assay could be deployed effectively in the regional decentralized laboratories nearest to HIV patients failing ART treatment, we assessed its usability, and the feasibility of performing the test by technicians with and without molecular laboratory experience (polymerase chain reaction (PCR)-experience) in Kenya. Separately, we tested archived samples from two studies [15,16] and remnant clinical samples from PLWH with viremia to evaluate its diagnostic accuracy. This validation assessed OLA-Simple’s “end-user” laboratory technician’s usability experience, as well as its performance characteristics in Kenya.

## 2. Methods

### 2.1. Study Design and Settings for OLA-Simple Validation

This was a prospective implementation and diagnostic validation study to evaluate the usability, feasibility, and diagnostic accuracy of OLA-Simple, with the aim of implementing the assay at decentralized laboratories near sites of patient care. The study was conducted between April and July 2022 in Kenya. The study comprised two complementary components: first, we assessed the usability and feasibility of performing OLA-Simple by laboratory technicians with different levels of molecular laboratory experience. This component evaluated the training requirements, ease of use, and user acceptability of the assay among PCR-experienced and PCR-inexperienced laboratory technicians. The second component evaluated the diagnostic accuracy of OLA-Simple for detecting selected HIV-1 DRMs in Kenya. This analysis utilized archived plasma samples from two prior clinical studies, and remnant samples, with HIV-DR Sanger sequencing results available from routine clinical monitoring tests for PLWH experiencing virological failure while on ART. OLA-Simple results were evaluated primarily against standard Sanger sequencing. However, to account for validly detected low-level variant mutations, a composite reference was employed. This reference incorporated HIV-DR results from Sanger sequencing (constituting the majority of the samples) together with tie-breaker testing, using the ELISA-based version of OLA that is capable of confirming low-frequency mutants (<20%). This composite reference is referred to here as “modified Sanger sequencing”.

OLA-Simple training and evaluation occurred at two centralized laboratories (also regarded as referral laboratories): the Kenya Medical Research Institute—Centre for Global Health Research (KEMRI-CGHR) HIV-Research Laboratory in western Kenya and the Nairobi at the National Public Health Laboratory (NPHL) in Nairobi. Both laboratory facilities had capacity for HIV PCR and Sanger DNA sequencing and were WHO-accredited regional laboratories for HIV-DR testing.

### 2.2. OLA-Simple Test Assay and Platform

The OLA-Simple test is designed as a near point-of-care oligonucleotide ligation assay for targeted detection of clinically relevant HIV-1 DRMs. An earlier version of the assay targeted five HIV-1 DRMs associated with resistance to first-line nucleos(t)ide reverse transcriptase inhibitor (NRTI) mutations, K65R and M184V, and non-nucleoside reverse transcriptase inhibitor (NNRTI) mutations, K103N, Y181C, and G190A [9]. For the present study, however, the test was optimized to include probes for two additional DRMs, L74V and Y115F, which are associated with NRTI abacavir resistance that is common among children living with HIV (CLHIV). The selection of these additional targets was guided by HIV-DRM data generated from the Sanger sequencing in the Opt4Kids study [15].

The OLA-Simple kit comprises lyophilized reagents for reverse transcription, PCR amplification, ligation, and lateral-flow detection mixes. These reagents were prepared and stabilized with additives, as previously described [12], and packaged in the Lutz Laboratory at the University of Washington, Seattle, WA, USA (picture shown in A of Supplementary Figure S1). The lyophilized reagents and the lateral flow detection strips for the OLA-Simple test were manufactured by the IEH Laboratories Consulting Group in Seattle, WA, USA except for the lyophilized reverse transcriptase mix that was prepared at the Lutz Laboratory. The RNA extraction reagents comprised a packaged pre-aliquoted QIAamp^®^ Viral RNA Mini Kit (Qiagen, Hilden, Germany).

For standardized assay execution and result interpretation, OLA-Simple testing was guided by Aquarium, a software application developed at the University of Washington, Seattle, WA, USA [9,10]. The application provided step-by-step instructions for conducting the test, including assay set-up, timing, and result interpretation. A flatbed scanner was integrated into the platform for the digitalization of interpretation of the visual images generated from lateral flow strips. The Aquarium digital guidance platform was installed on tablets and was used by the participating laboratory technicians throughout training, competency assessment, and clinical sample testing.

### 2.3. Laboratory Personnel and Classification

Laboratory technicians participating in this study were recruited from both referral testing laboratories and clinical patient-care hospital laboratories that supported HIV patient care or HIV-DR testing-related clinical studies. Specifically, these technicians had participated in the Optimizing Viral Suppression for Children (Opt4Kids) [15] and Optimizing Viral Suppression for Pregnant and Postpartum Women (Opt4Mamas) studies [16] or in routine HIV-DR testing activities.

For the purposes of this evaluation, the testing laboratory personnel were classified into two groups, based on their prior experience with molecular diagnostic tests. Five laboratory technicians working at the two centralized referral HIV-DR testing laboratories—KEMRI-CGHR HIV-Research Laboratory (2 technicians) and NPHL (3 technicians)—were classified as “PCR-experienced”. They were classified as such, given their routine involvement in PCR-based assays and Sanger sequencing to support national HIV-DR surveillance and clinical testing programs. These technicians were research technologists or scientists with university degree-level training in biomedical sciences. In contrast, eight laboratory technologists from four hospitals (two technicians from each) in western Kenya (Kisumu County Referral Hospital, Jaramogi Oginga Odinga Teaching and Referral Hospital, Ahero Sub-County Hospital and Nyakach Sub-County Hospital), who were not experienced in routinely performing PCR assays were regarded as “PCR-inexperienced”. These technicians primarily performed low-complexity routine clinical laboratory testing and held diploma-level or higher medical laboratory training. They did not have prior experience in performing PCR-based assays.

### 2.4. Training, Competency, and Implementation Workflow

The training and competency assessment for OLA-Simple were conducted in a standardized stepwise manner designed to evaluate the learnability and feasibility of the assay implementation in a laboratory setting. The OLA-Simple training program consisted of three sequential parts. First, the laboratory technicians received approximately four hours of virtual training, covering an overview of the principles of the OLA-Simple assay, interpretation of results, and a navigation of online Aquarium software application. The Aquarium software application provided step-by-step procedural guidance. Second, the laboratory technicians observed an in-person demonstration of the entire OLA-Simple workflow performed by a trained instructor, using two quality control (QC) samples. This session also provided the trainees with an opportunity for physical familiarization with the assay materials, as well as hands-on interaction with the digital guidance platform on Aquarium. Third, the trainee technicians underwent competency assessment by independently performing the first test on two QC samples while under direct observation by the instructor. Upon successful completion of the observed competency assessment, the technicians proceeded to perform an unobserved, independent testing of the same QC samples to confirm the reproducibility and operational readiness. Following completion of competency training, only PCR-experienced technicians advanced to perform blinded OLA-Simple testing of archived patient plasma samples.

**Figure 1.**
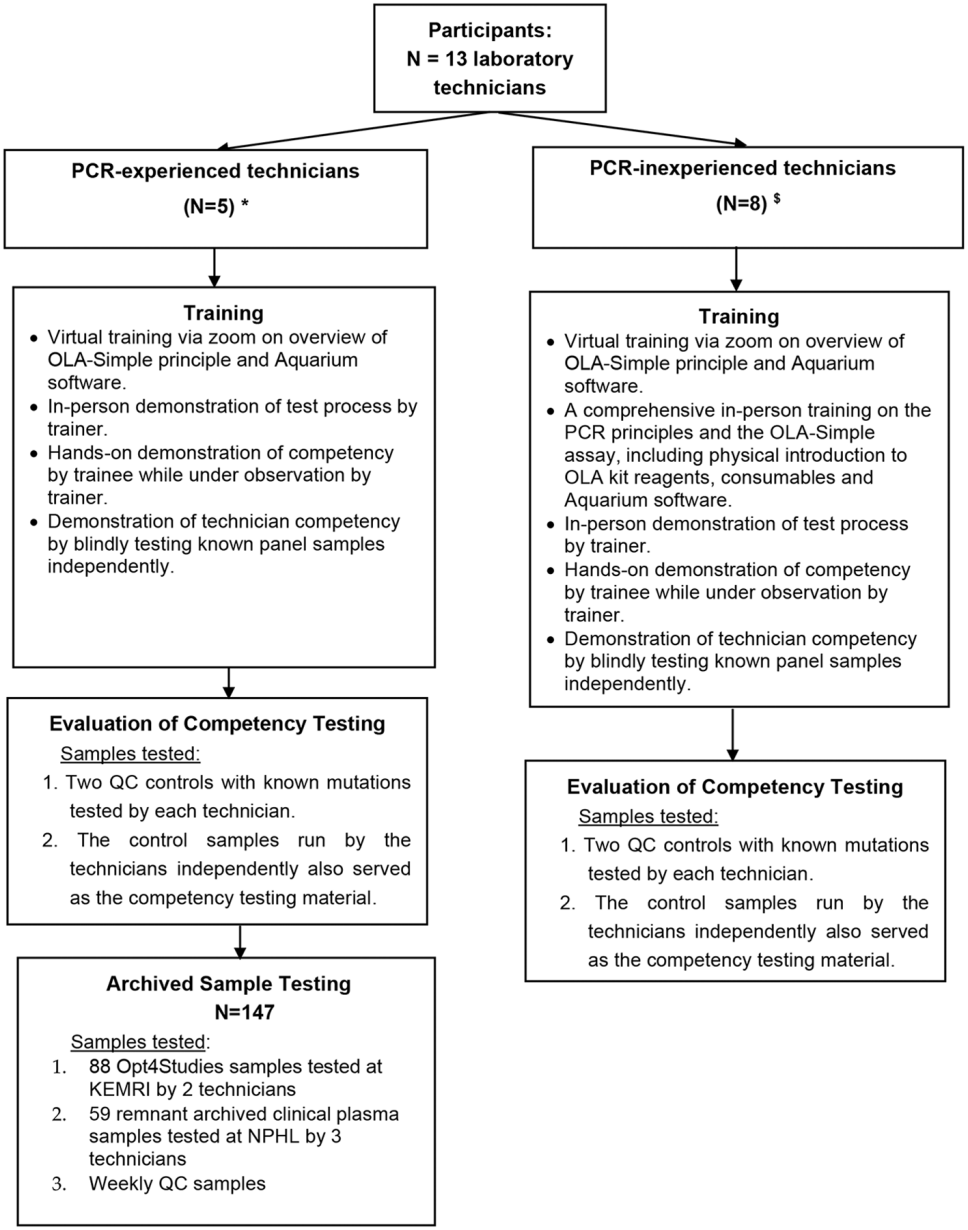
Diagrammatic representation of laboratory personnel training, competency, and testing schedule in the validation of the OLA-Simple assay for HIV-DR testing. This flowchart provides a diagrammatic representation of the laboratory personnel training, competency assessment, and testing involving technicians from various research institutions and hospital laboratories, who participated in the validation of OLA-Simple. The diagram indicates the number of PCR-experienced and PCR-inexperienced technicians and the training they received, and the competency testing and the testing of archived samples for the clinical in-field validation of OLA-Simple. Abbreviations: PCR, polymerase chain reaction; QC, quality control; NPHL, National Public Health Laboratory; Key: * National Public Health Reference Laboratory (N = 3) technicians; HIV Research Laboratory, KEMRI, Kisumu (N = 2) technicians. ^$^ Technicians from hospital-based diagnostic laboratories: Kisumu County Referral Hospital, Jaramogi Oginga Odinga Teaching and Referral Hospital, Nyakach Sub-County Hospital, and Ahero Sub-County Hospital.

PCR-inexperienced technicians participated in OLA-Simple training and competency assessment, utilizing quality control (QC) samples to assess the test’s learnability and ease-of-use and the feasibility of performing the test. They did not participate in testing of archived clinical patient samples, our second objective, due to their inability to be away from their regular workstations for a prolonged period (i.e., would have disrupted routine diagnostic services). Their participation in this study was, therefore, limited to training and competency testing, which we believe was sufficient to assess the usability and feasibility of OLA-Simple in the Kenyan level 4 or 5 clinical laboratory environment. The PCR-experienced technicians, in contrast, participated in both quality control sample testing and blinded testing of archived study and remnant clinical plasma samples that were previously genotyped. This latter group of technicians contributed to both the usability assessment and the diagnostic accuracy evaluation of OLA-Simple (Figure 1).

### 2.5. Technician Usability Framework and Evaluation

Usability of the OLA-Simple assay was evaluated by combining a structured question naire and direct observation of technician performance. Anonymous survey questionnaires were administered to the laboratory technicians before (pre-) and after (post-) training, to assess perceptions of usability before exposure and after completion of training and competency assessment.

The survey instruments were developed based on Nielsen’s usability framework, which evaluates five key domains: learnability, efficiency, memorability, error, and user satisfaction [17]. Each domain included multiple close-ended questions, assessed using Likert-scale responses, as well as open-ended questions to capture qualitative feedback on user experience, workflow challenges, and the perceived feasibility of implementing OLA-Simple in a routine laboratory setting. Definitions and example survey items of each usability domain are provided in Supplementary Table S1.

Post-training usability surveys were administered at different timepoints, depending on the technician’s role in the study. PCR-inexperienced technicians completed the post-training survey immediately following completion of competency testing, reflecting their experience with training and initial assay execution. On the other hand, PCR-experienced technicians completed the post-training survey at the end of study tests, when both competency assessment and blinded testing of archived plasma samples had been completed, allowing for the incorporation of feedback, based on extended hands-on use. Survey instruments and full questionnaires are provided in Supplementary Appendix S1.

### 2.6. Samples for the OLA-Simple Diagnostic Validation

**Figure 2.**
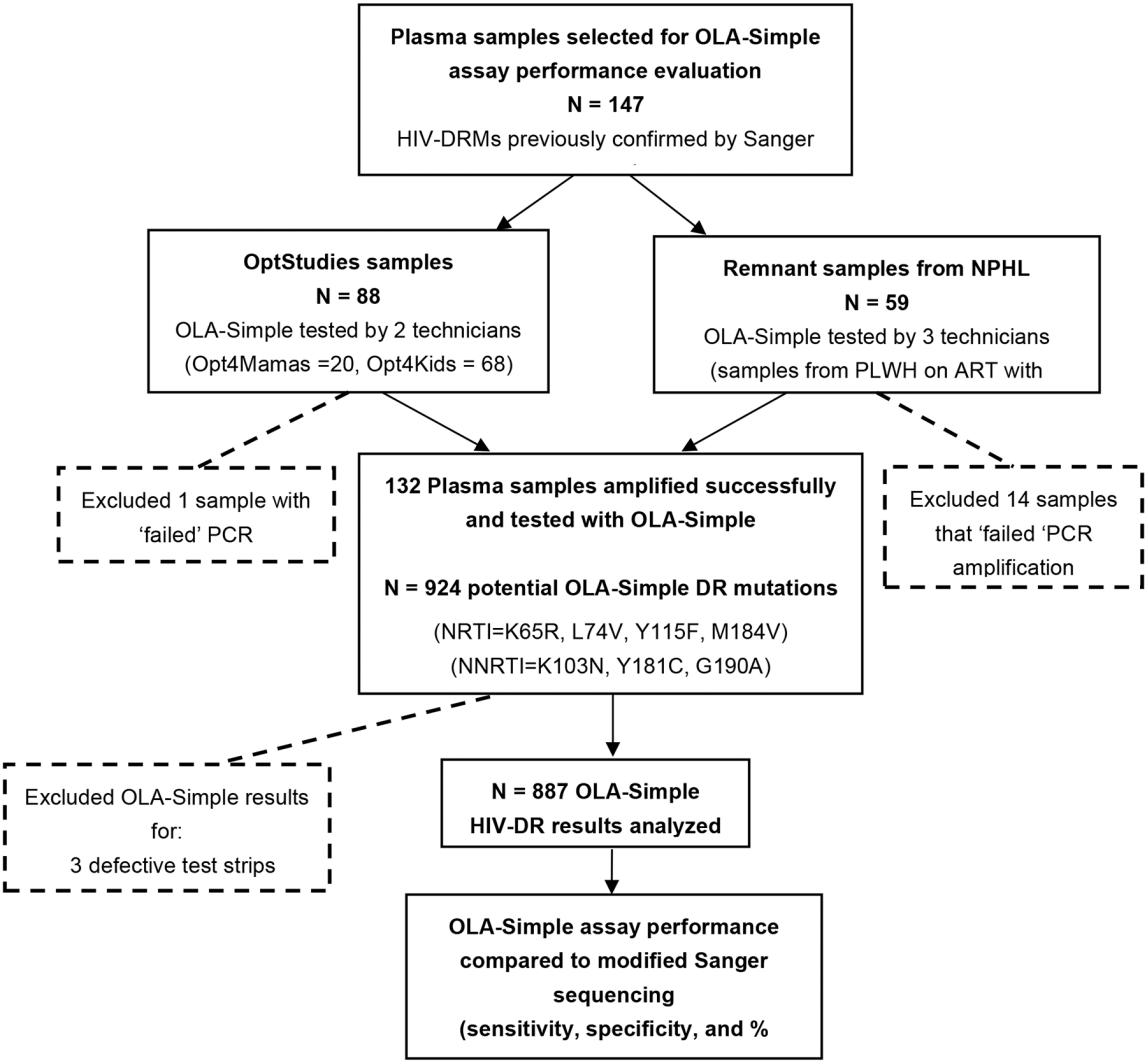
Flowchart of sample testing for validation of OLA-Simple compared to modified Sanger sequencing. Archived plasma samples from the OptStudies (Optimizing Viral Suppression in Children (Opt4Kids) and Pregnant and Postpartum Women (Opt4Mamas) studies), and from NPHL (National Public Health Laboratory) that were tested previously using Sanger sequencing, were selected for the validation of OLA-Simple. A total of 132 plasma samples were successfully amplified and tested with OLA-Simple for the 7 major potential HIV-DR mutations (that would yield 132 × 7 = 924 OLA-Simple point-mutation tests). We excluded 3 defective OLA-Simple strips and 34 indeterminate OLA-Simple results, therefore yielding a total of 887 OLA-Simple point-mutation results that were compared to analysis outcomes from modified Sanger sequencing. Abbreviations: HIV-DR, HIV drug resistance; PLWH, people living with HIV; PCR, polymerase chain reaction. Note: Given the greater sensitivity of OLA-Simple compared to Sanger sequencing’s primary reference, discordant results that are suggestive of a “false positive” were evaluated using a more sensitive semi-quantitative ELISA-based OLA tie-breaker assay. Of 26 discordant codons tested, five (four K103N and one M184V) were confirmed and therefore considered to be validly detected by OLA-Simple but not by Sanger sequencing. These five results (only) were substituted for the corresponding Sanger sequencing outcomes to generate the “modified Sanger sequencing” reference results.

Archived plasma samples from clinical HIV studies and remnant samples from routine monitoring of PLWH on ART with virologic failure that were previously characterized for HIV-DRMs by Sanger sequencing were used to evaluate the diagnostic accuracy of OLA-Simple in Kenya. A total of 147 de-identified plasma samples were selected and tested by PCR-experienced laboratory technicians. The samples were obtained from three sources: 68 plasma samples from the Opt4Kids study [15], conducted among CLHIV, and 20 archived plasma samples from the Opt4Mamas study [16], conducted among pregnant/postpartum women living with HIV. Together referred to as OptStudies samples, they were originally collected and genotyped as part of clinical research protocols. Lastly, 59 remnant archived plasma samples were obtained from PLWH-receiving ART who were documented as having virologic failure, defined as HIV-1 RNA viral load > 1000 copies/mL. These samples were collected between January 2019 and January 2022 from various parts of Kenya and sequenced for clinical detection of HIV-DR at NPHL for routine care and national surveillance activities. All these samples had previously been genotyped by Sanger sequencing for standard HIV-DRM detection, and they were de-identified prior to utilization for OLA-Simple validation. While OLA-Simple validation testing on the archived samples from the OptStudies were analyzed at the KEMRI-CGHR laboratory, the remnant archived routine clinical samples were tested at the NPHL. Sample selection and testing workflows for the diagnostic validation are summarized in Figure 2.

### 2.7. Quality Control Samples and Testing

Two QC samples with known DRM patterns were utilized throughout the study supporting training, competency testing, and quality assurance during testing of archived samples. The QC materials were prepared at the Lutz Laboratory (University of Washington), using mixtures of synthetic DNA gBlocks^®^ (Integrated DNA Technologies, Coralville, IA, USA) containing the HIV-DRMs targeted in this evaluation. The two QC samples contained a distinct combination of mutations: one with 30% K103N + 40% Y115F + 30% M184V +30% G190A and the second sample comprised 40% K65R + 60% L74V + 40% Y181C. These control samples were shipped to Kenya as lyophilized reagents and were resuspended on-site at 5000 copies/mL in HIV-negative plasma. These QC samples were tested by each of the Kenyan technicians during OLA-Simple training, competency testing, and on a weekly basis for approximately 10 weeks during clinical sample testing as part of a predefined quality assurance plan. The QC results were reviewed to assess technicians’ proficiency and consistency, and to monitor assay performance.

### 2.8. Sample Testing Procedures and Result Interpretation of OLA-Simple

OLA-Simple sample testing proceeded, using standardized procedures as previously described [12]. Briefly, viral RNA was extracted from plasma samples using the pre-aliquoted Qiagen Viral RNA Mini Kit reagents, following manufacturer instructions. As per the OLA-Simple protocol, the extracted RNA was reverse-transcribed in rehydrated lyophilized reverse-transcription reagents using a standard PCR thermocycler, and the complimentary DNA (cDNA) was subsequently PCR-amplified in rehydrated lyophilized PCR reagents. Next, aliquots of the amplified products were added to rehydrated lyophilized ligation reagent mixes that were specific for each mutation tested for probe ligation in a thermocycler. Ligation products were then applied to lateral flow detection strips in the presence of rehydrated colloidal gold particles. Lateral flow paper strips were allowed to develop for 15–20 min, scanned using a flatbed office scanner, and the digitalized images were uploaded into the Aquarium software platform to perform band visualization and analysis.

For each mutation assessed using a lateral flow strip, sample genotypes were classified as mutant (MUT), wild-type (WT), or indeterminate (IND), based on the intensity of genotype-specific bands, relative to predefined MUT and WT thresholds. For a lateral flow strip run to be considered valid, the control (CTRL) band was required to be present, along with a visible MUT and/or WT band. MUT thresholds were determined separately for each codon and for each study site, using control run results. Specifically, for a given codon at a given study site, the MUT threshold was defined using WT (0% MUT) control samples as the mean MUT band intensity plus three standard deviations (mean + 3 × SD). Samples with MUT band intensities exceeding the corresponding codon- and study-site-specific MUT threshold were classified as MUT. WT thresholds were determined independently for each study site using HIV-negative samples and were defined as the mean + 3 × SD of the WT band intensity measured in these HIV-negative samples. Within each study site, a single WT threshold was applied across all codons. Samples were classified as WT if they did not meet the MUT criteria and their WT band intensity exceeded the WT threshold. Lateral flow strips that neither met the WT nor MUT criteria but had the CTRL line intensity above the WT threshold were classified as IND. Lateral flow strips were classified as “defective” if the CTRL band was absent or if nonspecific markings interfered with the interpretation of the MUT or WT regions. Samples that failed to produce interpretable results across all seven mutations were classified as “failed amplification”. A diagrammatic representation of genotype band interpretation is shown in part B of Supplementary Figure S1.

### 2.9. Reference Standard and Tie-Breaker Testing

Diagnostic accuracy of OLA-Simple was mainly evaluated against Sanger sequencing as the reference standard. The samples included in this validation had been genotyped for the HIV-1 protease/reverse transcriptase gene-associated drug resistance previously, using the Applied Biosystems^™^ TaqPath^™^ Seq HIV-1 Genotyping Kit (Thermo Fisher Scientific, Waltham, MA, USA), which detects NRTI and NNRTI mutations at frequencies of approximately ≥ 20% viral quasispecies [5]. The Sanger sequencing analyses were performed at either KEMRI-CGHR or NPHL, depending on where samples had been referred earlier for testing.

As mentioned above, since OLA-Simple is capable of detecting low-frequency mutations below the limit of detection of consensus Sanger sequencing [18], additional testing was conducted to resolve discordant mutation results, which could “represent false positive” or “low-frequency” mutants. Remnant PCR amplicons generated during the OLA-Simple testing in Kenya were shipped to the Frenkel Laboratory (University of Washington, Seattle, WA, USA) and evaluated via the semi-quantitative, ELISA-based OLA method, previously validated against a next generation sequencing (NGS) platform [18]. The ELISA-based OLA employs the same ligation probes as OLA-Simple and uses mutation-specific standard curves to detect and semi-quantify mutant variants that are present at frequencies as low as 2% of the viral population [18]. Although simultaneous extraction and testing with high-sensitivity NGS platforms (e.g., Illumina or PacBio) would have been the ideal comparator, cost constraints precluded this approach. Therefore, ELISA-based OLA was used as a cost-effective alternative with sensitivity and specificity that was comparable to NGS methods for detecting low-frequency mutations below the detection threshold of Sanger sequencing.

Results from the ELISA-based OLA were used to adjudicate discordant findings between OLA-Simple and Sanger sequencing. Mutations detected by ELISA-based OLA but not by Sanger sequencing were considered to be true positives and were incorporated to constitute a composite reference standard, referred to in this analysis as “modified Sanger sequencing,” which was used for the primary diagnostic accuracy analyses.

### 2.10. Statistical Analysis

Descriptive characteristics of participating laboratory technicians were summarized using frequencies or medians as appropriate. Feedback on the usability survey, captured using Likert scales, was summarized as medians with interquartile ranges (IQRs).

For diagnostic accuracy analyses, OLA-Simple results were compared against modified Sanger sequencing as a reference standard. Mutation detection outcomes were summarized using frequencies and proportions. Additionally, in a sensitivity analysis, OLA-Simple performance was compared against the standard genotyping reference method—Sanger sequencing alone. OLA-Simple detection strips that were reported as defective or indeterminate were enumerated but excluded from diagnostic accuracy calculations. Diagnostic performance measures, including sensitivity, specificity, and percent agreement, were calculated with corresponding 95% confidence intervals (CIs), derived using the Clopper–Pearson method from 2 × 2 matrices comparing OLA-Simple against the modified Sanger sequencing results. All data wrangling and analysis were performed using R version 4.3.1 (R Project for Statistical Computing).

### 2.11. Ethics Approvals and Study Subjects’ Consent

Ethical approvals to use stored samples for OLA-Simple evaluation study were provided by the African Medical and Research Foundation (AMREF) and Jaramogi Oginga Odinga Teaching and Referral Hospital (JOOTRH) Institutional Review Boards (IRBs) in Kenya, as well as the University of Washington and the University of Colorado Denver IRBs in the USA. All adult study participants provided written informed consent, including laboratory technicians who participated in the OLA-Simple testing, and Opt4Mamas study participants. For the Opt4Kids study participants, however, written consent was obtained from their parents or legal guardians. Additionally, Opt4Kids children who were 13 years or older provided written assent. The use of de-identified archived samples from NPHL was part of the NPHL’s quality improvement activities and was considered to be exempt from ethics review.

## 3. Results

### 3.1. Laboratory Technician Participants, Training, and Usability Feedback

#### 3.1.1. Study Participants

A total of 13 laboratory technicians, five PCR-experienced and eight PCR-inexperienced technicians, participated in the OLA-Simple training (Table 1, Figure 1). Their median age was 33 years (interquartile range [IQR] 31, 34), five (38%) were female, nine (69%) had Bachelor’s or Master’s level university education, and only eight (61%) had ever performed a lateral flow strip test.

Competency assessment for the performance of the OLA-Simple test was performed by testing two QC samples by each laboratory technician and evaluated by scoring the presence or absence of the expected detection strip bands for each control sample. All five PCR-experienced laboratory technicians passed both observed and unobserved competency assessments with 100% accurate scores. Seven of the eight PCR-inexperienced laboratory technicians completed both observed and unobserved training and competency assessment, six passed with 100% accurate scores, and the seventh technician scored 86% due to the absence of bands in one detection strip (likely due to inadequate sample volume transfer). Thus, 12 of the 13 (92%) technician participants were deemed competent to perform OLA-Simple correctly and independently. Reasons for the one technician not completing competency training was because of unwillingness to continue with the independent competency testing process, which had seemed protracted.

#### 3.1.2. Laboratory Technician Usability Experience

Each of the seven PCR-inexperienced technicians who completed training tested at least four quality control samples, while the five PCR-experienced technicians tested at least 20 plasma samples from OptStudies samples (in addition to quality control samples), depending on the quantity of archived samples available at the two testing locations. OLA-Simple’s learnability, efficiency, and satisfaction domains were scored high by both PCR-experienced and -inexperienced laboratory technicians. The post-training survey feedback pertaining to learning, remembering, and running the test indicated that the technicians found the OLA-Simple attributes relatively “easy” and “comfortable”. The packaging of OLA-Simple reagents in modules and small convenient pouches, and guidance in the execution of the test utilizing Aquarium software were scored most favorably as being “helpful”. As a demonstration of the learnability ease, most of the technicians took >6 h to complete their first test during competency testing; however, testing time improved with experience and 80% of laboratory technicians could complete the test within six hours after two or more rounds of practice (Table 2).

As expected, the PCR-experienced technicians reported high comfort with the assay tasks and performed the assay procedures with relative ease within shorter turn-around-times (Table 2). Nonetheless, PCR-inexperienced technicians also reported ease of performing the assay highly. The PCR-experienced technicians were also more comfortable troubleshooting errors than PCR-inexperienced technicians. Having participated in more rounds of OLA-Simple testing for the validation portion of our study, the PCR-experienced participants indicated being comfortable running the OLA-Simple without entirely depending on step-by-step Aquarium software prompts, especially when executing sequential intuitive test steps. Sixty percent were comfortable with testing up to 6 samples in a single run batch using the OLA-Simple.

We observed overall improved scores in both technical skills and knowledge when we compared pre-versus post-training feedback among the laboratory technicians (Table 3). While comfort with micropipette usage was high at both pre- and post-training, familiarity with the PCR processes improved considerably post-training. Knowledge of PCR and HIV-DR, as well as technician confidence in handling smaller sample volumes, also improved.

All the laboratory technicians expressed that they would recommend OLA-Simple to their colleagues. Open-ended feedback in the surveys suggested that they found OLA-Simple “user-friendly and convenient,” “simple and amazing,” “fascinating,” “exciting,” and an “awesome HIV-DR test made simple.” Specifically, the laboratory technicians’ experiences with using dry reagents were reported as “simple and comfortable” and the majority found them “easy to manipulate.” The easy-to-reconstitute dry OLA-Simple reagents and the shortened sample-to-result test time were some of the features of the OLA-Simple HIV-DR test that were highly appreciated. Nonetheless, laboratory technicians had some suggestions for improvement. First, they suggested the need to create an offline version of the guiding software application (Aquarium) that could provide testing instructions even during times of poor internet connectivity. Second, they suggested the optimization of detection chemistry to eliminate the occasional faint background band on detection strips, especially on negative control samples (though they were correctly interpreted by the automated band-reading software). Third, the PCR-experienced participants found the Aquarium instructions to be tedious and to slow their execution once they had become familiar and comfortable with the assay procedures. Thus, they recommended including a simplified set of execution prompts that omitted sequential predictable steps in Aquarium for experienced users who were familiar with the assay protocol.

### 3.2. Assay Performance

#### 3.2.1. Outcomes of Testing Samples by OLA-Simple

A total of 147 plasma samples were tested by OLA-Simple that involved five PCR-experienced laboratory technicians at two HIV-DR testing laboratories. The OLA-Simple test results were compared to the modified Sanger sequencing results (Figure 2). Of the 147 samples tested, 132 (90%) of them were amplified and were tested by OLA-Simple. The successful OLA-Simple test consisted of 87 (66%) samples out of the 88 OptStudies samples [16,17] (analyzed by two technicians at KEMRI), and 45 (34%) out of the 59 archived remnant samples for routine HIV viral load testing (analyzed by three technicians at NPHL). The 132 successfully tested samples resulted in a total of 924 OLA-Simple detection strips (seven point-mutations per sample). Three strips (0.3%) were found to be defective (i.e., no CTRL line), and another 34 (3.7%) strips gave indeterminate OLA-Simple results (no MUT or WT signal above the cutoff value). These 37 strips were excluded from further analysis, resulting in 887 point-mutation detection strip results that were compared against the Sanger sequencing results (Figure 2).

#### 3.2.2. Performance of OLA-Simple

Analysis of the 887 detection strips yielded 271 (30.1%) HIV-DR mutations detected by OLA-Simple, as follows: 8 (2.9%) K65R; 25 (9.2%) L74VI; 11 (4.1%) Y115F; 77 (28.4%) K103N; 23 (8.5%) Y181C; 90 (33.2%) M184V; and 37 (13.7%) G190A. Discordant results (*n* = 26) that were OLA-Simple mutant positive (+) and Sanger sequencing mutant negative (*−*) were assessed by the tie-breaker, the semi-quantitative ELISA-plate-based OLA. Five mutations (four K103N and one M184V) were detected by the tie-breaker OLA, all present at < 20% frequencies. These five were deemed to be validly detected by OLA-Simple but not by Sanger sequencing. Thus, for the comparison of HIV-DR, these five tie-breaker results were reclassified by substituting the tie-breaker result for the Sanger sequencing only result, to constitute our “modified Sanger sequencing” reference result. The remaining 21 out of the 26 were resolved as discordant OLA-Simple results.

The OLA-Simple had an overall combined sensitivity of 97.3% (95% CI 94.5, 98.9), a specificity of 97.2% (95% CI 95.5, 98.3), and a percent agreement of 97.1% (95% CI 95.9, 98.2) across all seven mutations compared to modified Sanger sequencing (Table 4). OLA-Simple also detected each of the seven HIV-DRMs with high sensitivity, specificity, and percent agreements per mutation; all but one value was > 90%, as the specificity to K103N was 86.4% (95% CI 74.6, 93.8) (Table 4). Among the discordant results, 13/18 (72%) false positives by OLA-Simple were due to faint signal intensities in the region of the mutant band at or near the established detection threshold, which was the highest for the mutation K103N (*n* = 6). Seven false negatives by OLA-Simple were due mostly to the presence of alternative mutant variants that were not detected (*n* = 3) or HIV sequence polymorphisms that interfered with the probes’ ligation reaction and caused low band signal intensities (*n* = 2). The positive and negative predictive values for OLA-Simple for detection of each of the seven mutations were ≥75% and ≥94%, respectively, compared to modified Sanger sequencing (Table 5).

In our sensitivity analysis, when comparing the OLA-Simple results to Sanger sequencing only (excluding ELISA-based OLA), OLA-Simple detected each of the seven HIV-DRMs with high sensitivity, specificity, and percent agreement, ranging from 91.7% to 100%, 81.0% to 100%, and 94.7% to 100%, respectively (Table S2). Similarly, in the sensitivity analysis, the positive and negative predictive values for detecting each of the seven mutations were ≥ 75% and ≥94.7%, respectively (Table S3).

## 4. Discussion

This usability assessment of OLA-Simple among its intended users in Kenya demonstrates that this simplified, near-POC HIV-DR test can be performed after minimal training. All laboratory technician participants who completed the brief training demonstrated competency to perform the test, regardless of prior PCR experience or their laboratory training level. In addition, most technicians found OLA-Simple to be user-friendly and convenient. Further, the assay demonstrated high sensitivity and specificity compared to the current gold standard Sanger sequencing test. Overall, OLA-Simple results show high potential for future use at decentralized laboratories in LMIC settings.

The technicians’ feedback highlighted the convenience of the assay’s packaging that combined several PCR mix reaction ingredients into single-tube lyophilized reagents and had ensured that the assay’s reagents were optimally reconstituted with just one rehydration step. The OLA-Simple testing process was also found to be substantially faster than the standard of care Sanger sequencing HIV-DR analysis. Based on prior cost analysis, OLA-Simple developers estimate that the assay would be substantially less expensive than Sanger sequencing, as it does not require a DNA sequencer, uses fewer materials for testing, and has less stringent reagent storage requirements [19].

OLA-Simple demonstrated an overall sensitivity, specificity, and percent agreement of 97%, in detecting the targeted seven major point mutations tested in this validation. These mutations constituted the most clinically relevant viral genotypes associated with resistance to ART regimens among the patients. Unexpectedly, OLA-Simple demonstrated a weaker specificity (86%) for the K103N mutation than in past performance evaluation studies [11,12]. This was largely driven by the higher rates of false positive bands, due to background mutant signals that were not prominent in previous validation studies. The earlier ELISA-based OLA had reported satisfactory performance at other sites in a prospective trial involving Kenya samples [20]. Since the specificity was higher for the other codons, the OLA-Simple developers are confident that further technical optimization would improve the test performance of future OLA-Simple versions. Also, it is worth noting that the evaluated samples happened to have a few K65R point mutations that may have contributed in part to the low positive predictive value in our validation of the assay. Understandably, the inclusion of integrase strand transfer inhibitor (INSTI) resistance detection probes would have been of great relevance, considering the use of this drug class in current treatments in these settings. However, the specimens evaluated were collected prior to widespread use of INSTI drugs.

This validation highlights a few particularly important test qualities that make OLA-Simple well suited for future usability at decentralized laboratories in LMICs: specifically, its simplicity, rapid turnaround time, and accuracy [8]. The laboratory technicians rated OLA-Simple’s usability as “high”, including technicians who were PCR-inexperienced. This high usability score was probably driven by an appreciation of how lyophilized reagents simplified several molecular steps. Also, contributing to this score was likely the detection of mutations on lateral flow strips that were visually readable in most instances by the “naked eye,” as opposed to the gold standard Sanger sequencing, which requires the use of bioinformatics tools. Additionally, the step-by-step software-guided prompts facilitated ease of implementation. These user-friendly features of OLA-Simple’s earlier versions have also been noted by other investigators [8–10,12], though this is the first in-country, target-user evaluation. Additionally, OLA-Simple uses relatively inexpensive equipment, in contrast to other HIV-DR POC technologies [21–24].

The seven NRTI and NNRTI mutations selected for this OLA-Simple validation identified the “major” HIV-DR mutations conferring resistance to ARTs administered to children and adults living with HIV at the time the specimens were banked [15,16]. Now, such an evaluation would include detection for INSTI (e.g. dolutegravir) resistance mutations, which have been developed for OLA [25]. Thus, this evaluation stands as a proof-of-concept of how a near-POC HIV-DR test can support the detection of clinically meaningful DR, which would in turn support more personalized patient care in LMICs, such as has been performed with POC HIV-1 viral load testing for high-risk populations [26]. Second, OLA-Simple or related technologies can help us to meet the need of an anticipated multi-fold increase in HIV-DR testing in LMICs. Our related work has demonstrated that using a hub-and-spoke model for decentralizing HIV-DR testing in Kenya, using a near-POC technology, such as OLA-Simple, could not only decrease the turnaround time but also increase the processing rate fourfold [2]. While HIV-DR testing with OLA-Simple requires more advanced laboratory skills compared to HIV RNA quantification on more automated commercial platforms, lessons learned from decentralized near-POC HIV-1 viral load testing using platforms such as Cepheid’s GeneXpert may be applicable to HIV-DR testing [27]. Arguably, OLA-Simple uses common, simpler and relatively inexpensive laboratory equipment, including a thermocycler and a flatbed office scanner for the automation of result interpretation, compared to other POC HIV-DR technologies which require a real-time PCR platform for qPCR or suspension array system [21–24]. Nonetheless, each of these POC HIV-DR testing approaches, including OLA-Simple, comes with its own field implementation challenges, ranging from equipment installation and maintenance costs, technical skill set, to quality assurance plans, and thus, none fully meets the ASSURED (affordable, sensitive, specific, user-friendly, rapid, equipment-free, and deliverable) criteria endorsed by WHO [8]. Although this study was conducted in centralized laboratories—thereby benefiting from well-established biosafety measures and contamination controls—we believe that the primary objective of evaluating the usability and feasibility of OLA-Simple when operated by PCR-inexperienced technicians who did not routinely work in a molecular laboratory or with this assay, was not materially affected. OLA-Simple, as currently designed, could be implemented at decentralized laboratories with a stable power supply to run thermocyclers, where technicians are trained to pipette small material volumes, coupled with procedures and a strong quality assurance plan to prevent carryover contamination from the amplified samples. We envision that for OLA-Simple to move to a true POC technology, significant innovation in automation would be needed to achieve a similar capacity to some of the existing near-POC HIV-1 viral load systems, such as the GeneXpert^®^ (Cepheid, Sunnyvale, CA, USA) technology. Even then, none of the HIV-DR or viral load technologies are truly patient bedside.

This investigation has several strengths, including the in-country implementation of OLA-Simple as a near-POC assay, evaluation of its usability by Kenyan laboratory technicians, and the demonstration that a minimal set of molecular laboratory equipment is sufficient for it to generate clinically actionable HIV-DR results. Nonetheless, this work had several limitations. First, resource constraints prevented PCR-inexperienced technicians from testing clinical specimens. Second, the study testing was conducted in centralized molecular laboratories rather than in field settings where patient care occurs, which could have reduced the rigor of the analysis. Nevertheless, the primary objective was to assess trainability of both “novice” and “experienced” laboratory technicians in the OLA-Simple method, which we believe was not materially affected. Although cost analysis could have provided additional insight, it was beyond the scope of this study. Testing for INSTI (e.g., dolutegravir) resistance was also not performed, as relevant mutations were uncommon at the time the specimens were collected, as noted above. Like other near-POC HIV-DR assays in development, OLA-Simple detects specific “major” point-mutations [5]. Given the dynamic nature of HIV treatment, assay reagents will need to be updated to capture relevant resistance mutations for emerging ART classes. While dolutegravircontaining ART was initially considered highly effective, reports of resistance are increasing in LMICs [28]. In response, OLA-compatible reagents for major dolutegravir mutations have been developed and validated in the laboratory [25], enabling future implementation. Furthermore, our selection of only discrepant results for a tie-breaker analysis—often performed due to costs constraints—may have overestimated the sensitivity and specificity; future analyses should systematically evaluate the assay’s performance. Lastly, future analyses will prioritize the clinical relevance of OLA-Simple while aiming to increase the involvement of laboratory technicians from lower-cadre hospital facilities. Additionally, to further minimize potential low-level mutation bias, the use of an independent tie-breaker assay will be considered.

## 5. Conclusions

This implementation of OLA-Simple to detect HIV-DR in Kenya is a first of its kind in demonstrating the test’s high usability and test performance. Both PCR-experienced and PCR-inexperienced laboratory technician participants were able to successfully execute the OLA-Simple HIV-DR assay following brief training. The simplicity of the OLA-Simple reduces the turnaround-time relative to consensus sequencing, the standard method for HIV-DR testing, and could enable specimens to be tested in regional laboratories near patient-care sites in the LMICs. Tests such as OLA-Simple allow for country programs to strategize the use of near-POC HIV-DR assays to improve patient-level HIV treatment outcomes.

## Supplementary Material

Supplementary files

**Supplementary Materials:** The following supporting information can be downloaded at: https://www.mdpi.com/article/10.3390/laboratories3010005/s1, Appendix S1: Survey questionnaire completed pre- and pos-OLA-Simple training, Figure S1: Picture of OLA-Simple test detection kit content and a diagrammatic representation of the possible band combinations on detection strips. Table S1: Nielsen’s usability framework domains and example questions asked in our OLA-Simple usability survey, and Table S2: Sensitivity, specificity, and percent agreement of OLA-Simple (OS) compared to Sanger sequencing across drug resistance mutations (Table S2) and positive predictive value (PPV) and negative predictive value (NPV) of OLA-Simple (OS) compared to Sanger sequencing across drug resistance mutations (Table S3).

## Figures and Tables

**Table 1.** Pre-OLA-Simple training survey, (N = 13 laboratory technicians).

Variables	N (%) or Median (IQR^a^)
Primary language	
English	13 (100)
Kiswahili	0 (0)
Age (years)	33 (31, 34)
Female	5 (38)
Highest level of education	
Secondary, diploma, or the equivalent	3 (23)
Some university credit, no degree	1 (8)
Bachelor	8 (61)
Masters	1 (8)
Current occupation	
Medical lab technician/technologist	8 (62)
Research scientist/assistant	5 (38)
Years worked on current job	7 (6, 11)
Years worked in a clinical or research laboratory	7 (5, 8)
Prior usage of micropipette	13 (100)
Frequency of micropipette usage	
Daily	12 (92)
Weekly	1 (8)
Ever performed PCR assay with extraction of DNA or RNA from clinical samples (i.e., “PCR-experienced”)	5 (39)
Frequency of running a PCR assay (involving DNA/RNA handling)	
Daily	3 (23)
Weekly	1 (8)
Monthly	1 (8)
Ran PCR in the past but not currently	1 (8)
Never set up and run a PCR	7 (54)
Ever performed a lateral flow test	8 (62)
Frequency of running a lateral flow assay	
Daily	4 (31)
Monthly	1 (8)
Used in the past but not currently	3 (23)
Never performed a lateral flow test	5 (39)
Awareness of the need for pre-and post-PCR area separation in a lab performing PCR	12 (92)
Feasible to have pre/post PCR area in your current lab	8 (62)
Comfortability with micropipette use	5 (4, 5) *
Comfortability with pipetting small volumes	4 (4, 5) *
Understanding of PCR principles	4 (3, 5) *
Familiarity with how PCR works	3 (2, 5) *
Understanding of HIV drug resistance	4 (3, 5) *
Comfortability of explaining mutations	4 (2, 5) *

a IQR: Interquartile range

* The median and IQR survey feedback represented Likert scores that ranged between 1 and 5, whereby 1 represented “not comfortable/not familiar/do not understand at all”, and 5 represented “very comfortable/familiar/understand very well”.

**Table 2.** Post-OLA-Simple training survey (N = 12^$^ laboratory technicians).

Variables	Total Group (N = 12) n(%) or Median (IQR)	PCR-Inexperienced (N = 7) n(%) or Median (IQR)	PCR-Experienced (N = 5) n(%) or Median (IQR)
Length of time to run OLA-Simple for first time			
5 hours	2 (17)	1 (14)	1 (20)
6 hours	4 (33)	2 (29)	2 (40)
7 hours or more	6 (50)	4 (57)	2 (40)
Length of time to run OLA-Simple after first time			
4 hours	4 (33)	2 (29)	2 (40)
5 hours	4 (33)	3 (43)	1 (20)
6 hours	2 (17)	1 (14)	1 (20)
7 hours or more	2 (17)	1 (14)	1 (20)
Number of tests run in 1 day	4 (2, 5.75)	4 (2, 4)	8 (3, 12)
Comfortable number of samples running simultaneously			
2 samples	4 (33)	4 (57)	0
3 samples	1 (8)	1 (14)	0
4 samples	4 (33)	2 (29)	2 (40)
5 or more samples	3 (25)	0	3 (60)
Ease of learning OLA-Simple	5 (4, 5)	4 (4, 5)	5 (5, 5)
Ease of remembering procedure	4 (4, 5)	4 (4, 4.5)	5 (4, 5)
Ease of running OLA-Simple after a period of absence	4.5 (3, 5)	4 (3, 4.5)	5 (5, 5)
Comfortability of running procedure without Aquarium software	4 (2, 5)	2 (1.5, 4)	4 (4, 5)
Helpfulness of Aquarium software	5 (4, 5)	5 (4.25, 5)	4 (4, 5)
Difficulty finding correct reagent pouch	5 (4, 5)	5 (3.5, 5)	5 (5, 5)
Clearness of OLA-Simple reagent labeling system	5 (5, 5)	5 (5, 5)	5 (5, 5)
Helpfulness of OLA-Simple color matching system	5 (5, 5)	5 (5, 5)	5 (5, 5)
Helpfulness of OLA-Simple barcode system	5 (4.75, 5)	5 (5, 5)	5 (4, 5)
Number of lines comfortable interpreting on lateral flow test			
3 lines	1 (8)	1 (14)	0
4 lines	5 (42)	3 (43)	2 (40)
5 lines	5 (42)	3 (43)	2 (40)
6 lines	0	0	0
7 or more lines	1 (8)	0	1 (20)
Comfortability with micropipette use	5 (5, 5)	5 (5, 5)	5 (5, 5)
Comfortability with pipetting small volumes	5 (5, 5)	5 (5, 5)	5 (5, 5)
Understanding of PCR principles	5 (4, 5)	4 (3.5, 5)	5 (5, 5)
Familiarity with how PCR works	5 (3.75, 5)	4 (3, 5)	5 (5, 5)
Understanding of HIV drug resistance	5 (4, 5)	4 (3.5, 4.5)	5 (5, 5)
Comfortability of explaining mutations	4.5 (4, 5)	4 (4, 4.5)	5 (5, 5)
Able to continue after error occurred	3 (25)	0 (0)	3 (60)
Would recommend to colleagues	12 (100)	7 (100)	5 (100)

IQR: Interquartile range; the median and IQR survey feedback represented Likert scores that ranged between 1 and 5, whereby 1 represented “not comfortable/very difficult/not useful” and 5 represented “very comfortable/very easy/very useful”;

$ one technician did not complete the training and therefore was not included in the post-training survey.

Note: The post-training survey feedback was submitted after completion of sample testing; for the PCR-naïve, it followed two rounds of competency assessment using control samples while for the PCR-experienced technicians, it took place after multiple rounds of testing archived clinical samples following their competency testing.

**Table 3.** Comparison of pre- versus post-training understanding and familiarity with assay procedures (N = 12 laboratory technicians).

Variable	Pre-Training N = 13 Median (IQR)	Post-Training N = 12 * Median (IQR)
Comfortability with micropipette use	5 (4, 5)	5 (5, 5)
Comfortability with pipetting small volumes	4 (4, 5)	5 (5, 5)
Understanding of PCR principles	4 (3, 5)	5 (4, 5)
Familiarity with how PCR works	3 (2, 5)	5 (3.75, 5)
Understanding of HIV drug resistance	4 (3, 5)	5 (4, 5)
Comfortability of explaining mutations	4 (2, 5)	4.5 (4, 5)

IQR: Interquartile range; the median and IQR survey feedback represented Likert scores that ranged between 1 and 5, whereby 1 represented “not comfortable/do not understand at all” and 5 represented “very comfortable/understand very well”;

* PCR-inexperienced technician who did not complete competency testing has been excluded.

**Table 4.** Sensitivity, specificity, and percent agreement for OLA-Simple (OS) compared to modified Sanger sequencing across drug resistance mutations.

	Mutation Detected by OS/Modified Sanger Sequencing *		Wild-Type Codon by OS/Modified Sanger Sequencing		Percent Agreement (95% CI)
Resistance Mutation	n/N (*n* = True Positives by OS (TP), N = Actual Positives by Modified Sanger Sequencing (TP + FN))	Sensitivity (95% CI)	n/N (*n* = True Negatives by OS (TN), N = Actual Negatives by Modified Sanger Sequencing (TN + FP))	Specificity (95% CI)	
K65R	6/6	100% (54.1, 100)	114/116	98.3% (94.0, 99.8)	98.4% (94.2, 99.8)
L74VI	25/25	100% (86.3, 100)	101/101	100% (96.4, 100)	100% (97.1, 100)
Y115F	11/12	91.7% (61.5, 99.8)	114/114	100% (96.8, 100)	99.2% (95.6, 99.9)
M184V	88/90	97.8% (92.2, 99.7)	36/38	94.7% (82.2, 99.3)	96.9% (92.2, 99.1)
K103N	69/71	97.2% (88.3, 99.1)	51/59	86.4% (74.6, 93.8)	91.5% (85.3, 95.7)
Y181C	21/22	95.5% (77.1, 99.9)	104/106	98.1% (93.3, 99.8)	97.7% (93.3, 99.5)
G190A	33/34	97.1% (84.7, 99.9)	89/93	95.7% (89.3, 98.8)	96.1% (91.0, 98.7)
**Total n/N**	**253/260**	**97.3% (94.5, 98.9)**	**609/627**	**97.2% (95.5, 98.3)**	**97.1% (95.9, 98.2)**

* Modified Sanger sequencing indicates HIV-DRMs results with Sanger sequencing in conjunction with semi-quantitative ELISA-based OLA; Abbreviations: OS: OLA-Simple; TP: true positive; TN: true negative; and n/N represent OLA-Simple results/modified Sanger sequencing results.

**Table 5.** Positive predictive value (PPV) and negative predictive value (NPV) for OLA-Simple (OS) compared to modified Sanger sequencing across drug resistance mutations.

Resistance Mutation	Positive Values		Negative Values	
	n/N (n = True Positives by OS (TP), N = Positives by Modified Sanger sequencing* (TP + FP))	PPV	n/N (n = True Negatives by OS (TN), N = Negatives by Modified Sanger sequencing* (TN + FN))	NPV
K65R	6/8	75.0%	114/114	100%
L74VI	25/25	100%	101/101	100%
Y115F	11/11	100%	114/115	99.1%
M184V	88/90	97.8%	36/38	94.7%
K103N	69/77	89.6%	51/53	96.2%
Y181C	21/23	91.3%	104/105	99.0%
G190A	33/37	89.2%	89/90	98.9%

* Modified Sanger sequencing indicates HIV-DRMs results with Sanger sequencing in conjunction with semi-quantitative ELISA-based OLA; Abbreviations: OS: OLA-Simple; TP: true positive; FP: false positive; TN: true negative; FN: false negative; PPV: positive predictive value; NPV: negative predictive value; and n/N represent OLA-Simple results/modified Sanger sequencing results

## Data Availability

The study data are available upon request to the corresponding author.

## Abbreviations

The following abbreviations have been used in this manuscript:

POC: Point-of-care
HIV-DR: Human immunodeficiency virus drug resistance
LMICs: Low- and middle-income countries
OLA: Oligonucleotide ligation assay
PCR: Polymerase chain reaction
ART: Antiretroviral therapy/treatment
PLWH: People living with HIV
ELISA: Enzyme-linked immunosorbent assay
KEMRI: Kenya Medical Research Institute
NPHL: National Public Health Laboratories (in Kenya)
CLHIV: Children living with HIV
NRTI: Nucleos(t)ide reverse transcriptase
NNRTI: Non-nucleoside reverse transcriptase
INSTI: Integrase strand transfer inhibitor
NGS: Next generation sequencing
WT: Wild-type genotype
MUT: Mutant genotype
IND: Indeterminate genotype
CTRL: Control

## References

[1] HIV Drug Resistance—Brief Report 2024. Available online: https://www.who.int/publications-detail-redirect/9789240086319 (accessed on 29 April 2024).

[2] Wang Y; Kingwara L; Wagner AD; Yongo N; Hassan SA; Liu S; Oyaro P; Patel RC Optimising HIV drug resistance testing laboratory networks in Kenya: Insights from systems engineering modelling. BMJ Open 2024, 14, e079988. 10.1136/bmjopen-2023-079988 PMC11146353 38569688

[3] Manyana S; Gounder L; Pillay M; Manasa J; Naidoo K; Chimukangara B HIV-1 Drug Resistance Genotyping in Resource Limited Settings: Current and Future Perspectives in Sequencing Technologies. Viruses 2021, 13, 1125. 34208165 10.3390/v13061125 PMC8230827

[4] Kantor R Next Generation Sequencing for HIV-1 Drug Resistance Testing—A Special Issue Walkthrough. Viruses 2021, 13, 340. 33671700 10.3390/v13020340 PMC7926934

[5] Parkin N; Harrigan PR; Inzaule S; Bertagnolio S Need assessment for HIV drug resistance testing and landscape of current and future technologies in low- and middle-income countries. PLoS Glob. Public Health 2023, 3, e0001948. 37851634 10.1371/journal.pgph.0001948 PMC10584185

[6] Noguera-Julian M HIV drug resistance testing—The quest for Point-of-Care. eBioMedicine 2019, 50, 11–12. 31810819 10.1016/j.ebiom.2019.11.040 PMC6921291

[7] da Silva J; Pals S; Chang J; Hackett S; Godfrey C; Raizes E Monitoring Emerging Human Immunodeficiency Virus Drug Resistance in Sub-Saharan Africa in the Era of Dolutegravir. J. Infect. Dis 2022, 225, 364–366. 34282844 10.1093/infdis/jiab382

[8] Chua RJ; Capiña R; Ji H Point-of-Care Tests for HIV Drug Resistance Monitoring: Advances and Potentials. Pathogens 2022, 11, 724. 35889970 10.3390/pathogens11070724 PMC9321160

[9] Panpradist N; Beck IA; Vrana J; Higa N; McIntyre D; Ruth PS; So I; Kline EC; Kanthula R; Wong-On-Wing A; OLA-Simple: A software-guided HIV-1 drug resistance test for low-resource laboratories. EBioMedicine 2019, 50, 34–44. 31767540 10.1016/j.ebiom.2019.11.002 PMC6921160

[10] Vrana J; de Lange O; Yang Y; Newman G; Saleem A; Miller A; Cordray C; Halabiya S; Parks M; Lopez E; Aquarium: Open-source laboratory software for design, execution and data management. Synth. Biol 2021, 6, ysab006. 10.1093/synbio/ysab006 PMC8209617 34151028

[11] Panpradist N; Beck IA; Chung MH; Kiarie JN; Frenkel LM; Lutz BR Simplified Paper Format for Detecting HIV Drug Resistance in Clinical Specimens by Oligonucleotide Ligation. PLoS ONE 2016, 11, e0145962. 26751207 10.1371/journal.pone.0145962 PMC4713472

[12] Panpradist N; Beck IA; Ruth PS; Ávila-Ríos S; García-Morales C; Soto-Nava M; Tapia-Trejo D; Matías-Florentino M; Paz-Juarez HE; Del Arenal-Sanchez S; Near point-of-care, point-mutation test to detect drug resistance in HIV-1: A validation study in a Mexican cohort. AIDS Lond. Engl 2020, 34, 1331–1338. 10.1097/QAD.0000000000002524 PMC8284934 32205723

[13] Vrana JD; Panpradist N; Higa N; Ko D; Ruth P; Kanthula R; Lai JJ; Yang Y; Sakr SR; Chohan B; Implementation of an interactive mobile application to pilot a rapid assay to detect HIV drug resistance mutations in Kenya. PLoS Glob. Public Health 2022, 2, e0000185. 36962187 10.1371/journal.pgph.0000185 PMC10021139

[14] National Syndemic Disease Control Council—NSDCC. National Syndemic Disease Control Council. Available online: https://nsdcc.go.ke/ (accessed on 29 April 2024).

[15] Abuogi L; Oyaro P; Wakjira G; Thomas KK; Scallon AJ; Mukui I; Chohan BH; Brown E; Karauki E; Yongo N; HIV Drug Resistance Patterns and Characteristics Associated with Clinically Significant Drug Resistance among Children with Virologic Failure on Antiretroviral Treatment in Kenya: Findings from the Opt4Kids Randomized Controlled Trial. Viruses 2023, 15, 2083. 37896860 10.3390/v15102083 PMC10612029

[16] Patel RC; Oyaro P; Thomas KK; Basha GW; Wagude J; Mukui I; Brown E; Hassan SA; Kinywa E; Oluoch F; Impact of point-of-care HIV viral load and targeted drug resistance mutation testing on viral suppression among Kenyan pregnant and postpartum women: Results from a prospective cohort study (Opt4Mamas). J. Int. AIDS Soc 2023, 26, e26182. 37938856 10.1002/jia2.26182 PMC10631517

[17] Usability Attribute—An Overview|ScienceDirect Topics. Available online: https://www.sciencedirect.com/topics/computer-science/usability-attribute (accessed on 15 April 2024).

[18] Beck IA; Deng W; Payant R; Hall R; Bumgarner RE; Mullins JI; Frenkel LM Validation of an oligonucleotide ligation assay for quantification of human immunodeficiency virus type 1 drug-resistant mutants by use of massively parallel sequencing. J. Clin. Microbiol 2014, 52, 2320–2327. 24740080 10.1128/JCM.00306-14 PMC4097683

[19] Duarte HA; Babigumira JB; Enns EA; Stauffer DC; Shafer RW; Beck IA; Garrison LP; Chung MH; Frenkel LM; Bendavid E Cost-effectiveness analysis of pre-ART HIV drug resistance testing in Kenyan women. EClinicalMedicine 2020, 22, 100355. 32490370 10.1016/j.eclinm.2020.100355 PMC7256304

[20] Chung MH; McGrath CJ; Beck IA; Levine M; Milne RS; So I; Andersen N; Dross S; Coombs RW; Chohan B; Evaluation of the management of pretreatment HIV drug resistance by oligonucleotide ligation assay: A randomised controlled trial. Lancet HIV 2020, 7, e104–e112. 31818716 10.1016/S2352-3018(19)30337-6 PMC6936934

[21] MacLeod IJ; Rowley CF; Essex M PANDAA intentionally violates conventional qPCR design to enable durable, mismatchagnostic detection of highly polymorphic pathogens. Commun. Biol 2021, 4, 227. 33603155 10.1038/s42003-021-01751-9 PMC7892852

[22] Zhang G; Cai F; de Rivera IL; Zhou Z; Zhang J; Nkengasong J; Gao F; Yang C Simultaneous Detection of Major Drug Resistance Mutations of HIV-1 Subtype B Viruses from Dried Blood Spot Specimens by Multiplex Allele-Specific Assay. J. Clin. Microbiol 2016, 54, 220–222. 26560533 10.1128/JCM.02833-15 PMC4702749

[23] Kouamou V; Manasa J; Katzenstein D; McGregor AM; Ndhlovu CE; Makadzange T Diagnostic Accuracy of Pan-Degenerate Amplification and Adaptation Assay for HIV-1 Drug Resistance Mutation Analysis in Low- and Middle-Income Countries. J. Clin. Microbiol 2020, 58, e01045–20. 32522826 10.1128/JCM.01045-20 PMC7448631

[24] Zhang L; Wang J; Coetzer M; Angione S; Kantor R; Tripathi A One-Step Ligation on RNA Amplification for the Detection of Point Mutations. J. Mol. Diagn 2015, 17, 679–688. 26322949 10.1016/j.jmoldx.2015.07.001 PMC4630173

[25] Beck IA; Boyce CL; Bishop MD; Vu YL; Fung A; Styrchak S; Panpradist N; Lutz BR; Frenkel LM Development and Optimization of Oligonucleotide Ligation Assay (OLA) Probes for Detection of HIV-1 Resistance to Dolutegravir. Viruses 2024, 16, 1162. 39066324 10.3390/v16071162 PMC11281587

[26] Giang JH; Basha G; Thomas KK; Oyaro P; Chohan BH; Kingwara L; Hassan SA; Yongo N; Wagude J; Oluoch F; Real-world performance of point-of-care vs. standard-of-care HIV viral load testing in western Kenya: Secondary analysis of Opt4Kids and Opt4Mamas studies. PLoS Glob. Public Health 2024, 4, e0003378. 38913630 10.1371/journal.pgph.0003378 PMC11195974

[27] Wang Y; Wagner AD; Liu S; Kingwara L; Oyaro P; Brown E; Karauki E; Yongo N; Bowen N; Kiiru J; Using queueing models as a decision support tool in allocating point-of-care HIV viral load testing machines in Kisumu County, Kenya. Health Policy Plan. 2024, 39, 44–55. 37949109 10.1093/heapol/czad111 PMC10775219

[28] Fokam J; Inzaule S; Colizzi V; Perno C-F; Kaseya J; Ndembi N HIV drug resistance to integrase inhibitors in low- and middle-income countries. Nat. Med 2024, 30, 618–619. 38263265 10.1038/s41591-023-02763-0 PMC11586446

