# Supplementary material for: Oligonucleotide Ligation Assay (OLA)-Simple: Field Implementation, Usability, and Performance of a near Point-of-Care HIV Drug Resistance Assay in Kenya
Source: Laboratories. Author manuscript; Available in PMC 2026 Apr 3. (PMC13046437; doi:10.3390/laboratories3010005)
Supplement: Supplementary files [file NIHMS2159182-supplement-Supplementary_files.zip › Appendix_S1.pdf]

Pre-OLA Simple Training

Please complete the survey below.Thank you!

Study ID	__________________________________
Date	__________________________________

Section 1 Background

What is your primary language?	◯ English<br>◯ Kiswahili<br>◯ Dholuo<br>◯ Other
How old are you today in years?	__________________________________
What is your highest level of education?	◯ Completed secondary school, diploma or the equivalent<br>◯ Some university credit, no degree<br>◯ Trade/technical/vocational training<br>◯ Associate's<br>◯ Bacherlor's<br>◯ Master's<br>(change the choices according to Bhavna)
What is your current occupation?	__________________________________
How many years have you been working in this occupation?	__________________________________
How many total years have you worked in a clinical or research laboratory setting?	__________________________________
Briefly describe what you do in your current position	__________________________________

Section 2 Current Knowledge and Training

Do you have experience performing molecular tests?	◯ Yes<br>◯ No
Describe what tests you have performed:	__________________________________
Have you used a micropipette ever before?	◯ Yes<br>◯ No
How often do you use a micropipette?	◯ Daily<br>◯ Weekly<br>◯ Monthly<br>◯ Less frequent than monthly<br>◯ Used in the past but not currently

Have you ever performed extraction of DNA or RNA from clinical samples?	◯ Yes<br>◯ No
What kit did you use?	__________________________________
Have you ever set up and run a PCR?	◯ Yes<br>◯ No
How often do you run a PCR?	◯ Daily<br>◯ Weekly<br>◯ Monthly<br>◯ Less frequent than monthly<br>◯ Ran PCR in the past but not currently
Have you performed a lateral flow test ever before?	◯ Yes<br>◯ No
What type of lateral flow tests have you run before?	__________________________________
How often do you run a lateral flow test?	◯ Daily<br>◯ Weekly<br>◯ Monthly<br>◯ Less frequent than monthly<br>◯ Used in the past but not currently
Are you aware of the need for pre- and post-PCR area separation in a lab performing PCR?	◯ Yes<br>◯ No
How feasible is it to establish a pre- and post-PCR area in your current lab? In other words, does your laboratory have access to two physically separated areas or is there space that can be divided into two separated areas if needed? Please explain.	__________________________________

On a scale of 1-5, where 1 is not comfortable/don't understand at all, and 5 is very comfortable/understand very well:

	1 not comfortable/ don't understand at all	2	3	4	5 very comfortable/ understand very well
How comfortable are you using a micropipetter?	◯	◯	◯	◯	◯
How comfortable are you pipetting a small volume (such as 3uL or less)?	◯	◯	◯	◯	◯
How well do you understand the basic principles of a PCR?	◯	◯	◯	◯	◯

How familiar are you with how PCR works (such as the theoretical components, which include the dNTPs, primers, the polymerase, or extension)?	○	○	○	○	○
How well do you understand how HIV drug resistance mutations occur?	○	○	○	○	○
How comfortable would you feel explaining what a mutation like K103N indicates?	○	○	○	○	○

Post-OLA Simple Training

Please complete the survey below.Thank you!

Email ________________________

Date ________________________

Section 1 Overall Experience

What was your overall experience using the OLA Simple kit? ________________________

What was your experience using pre-made, dried reagents in OLA Simple? ________________________

How long did it take to run OLA Simple for the first time?
- ◯ 4 hours
- ◯ 5 hours
- ◯ 6 hours
- ◯ 7 hours or more

After learning how to run OLA Simple the first time, how long does it usually take you to run it once?
- ◯ 4 hours
- ◯ 5 hours
- ◯ 6 hours
- ◯ 7 hours or more

How many tests can you do in one day? ________________________

How many samples would you feel comfortable running simultaneously at the same time?
- ◯ 1
- ◯ 2
- ◯ 3
- ◯ 4
- ◯ 5 or more

On a scale of 1-5, where 1 is not comfortable/very difficult/not useful and 5 is very comfortable/very easy/very useful:

	1 is not comfortable / very difficult / not useful	2	3	4	5 is very comfortable / very easy / very useful
How easy was OLA Simple to learn?	◯	◯	◯	◯	◯
Was the procedure intuitive and easy to remember?	◯	◯	◯	◯	◯
After a period of absence of one month, how easy would it be to run OLA Simple again?	◯	◯	◯	◯	◯

How comfortable would you feel running the procedure without Aquarium software and only written instructions?	◯	◯	◯	◯	◯
How useful was the interactive Aquarium software protocol in assisting you to carry out the work flow?	◯	◯	◯	◯	◯
How difficult was finding each of the packages referenced in the OLA Simple protocol?	◯	◯	◯	◯	◯
How clear was the OLA Simple reagent labelling system?	◯	◯	◯	◯	◯
How helpful was the OLA Simple color matching system?	◯	◯	◯	◯	◯
How helpful was the OLA Simple barcode system for the recognition of materials in the kit?	◯	◯	◯	◯	◯

Section 2 Current Knowledge and Training

In your opinion, why is it necessary to separate pre- and post-PCR lab areas? ________________________________

What does each line on the lateral flow strip indicate? ________________________________

If we added more lines on a single lateral flow test in the future, how many lines would you feel comfortable interpreting?

◯ 3
◯ 4
◯ 5
◯ 6
◯ 7 or more

How could we make the lateral flow results easier to visualize or analyze? For example, any thoughts on improving visual recognition of the drug resistance bands or automating detection? ________________________________

On a scale of 1-5, where 1 is not comfortable/don't understand at all, 5 is very comfortable/understand very well:

	1 is not comfortable / don't understand at all	2	3	4	5 is very comfortable / understand very well
How comfortable are you using a micropipetter?	◯	◯	◯	◯	◯

How comfortable are you pipetting a small volume (such as 3uL or less)?	○	○	○	○	○
How well do you understand the basic principles of a PCR?	○	○	○	○	○
How familiar are you with how PCR works (such as the theoretical components, which include the dNTPs, primers, the polymerase, or extension)?	○	○	○	○	○
How well do you understand how HIV drug resistance mutations occur?	○	○	○	○	○
How comfortable would you feel explaining what a mutation like K103N indicates?	○	○	○	○	○

Section 3 Feedback

Which OLA Simple protocol instruction(s) were the hardest to follow? Please explain why.	__________________________________
What suggestions do you have on how to make the OLA Simple v.1 kit and instructions easier to use?	__________________________________
Did you encounter any errors while running OLA Simple?	○ Yes<br>○ No
What error(s) occurred and how could they be avoided in the future?	__________________________________
Were you able to continue the same test after the error or did you have to start over?	○ Continued same test<br>○ Started over
Can you identify any areas where errors could easily be made by you that would require you to start over?	__________________________________
What did you like the most about using the OLA-Simple kit?	__________________________________
What did you dislike the most about using the OLA Simple kit?	__________________________________
What did you learn from participating in this training?	__________________________________
Do you think other technicians would benefit from participating in the OLA Simple training session? Why or why not?	__________________________________<br>(can also make this yes/no)
Would you recommend this activity to your colleagues?	○ Yes<br>○ No

Do you have any other suggestions on how to improve OLA Simple, its Aquarium software, its protocol, or its training for future use?	__________________________________
Would it be okay to use photos of our training sessions that include you in OLA Simple dissemination, including on websites or in conference presentations?	◯ Yes<br>◯ No
