# Supplementary material for: Oligonucleotide Ligation Assay (OLA)-Simple: Field Implementation, Usability, and Performance of a near Point-of-Care HIV Drug Resistance Assay in Kenya
Source: Laboratories. Author manuscript; Available in PMC 2026 Apr 3. (PMC13046437; doi:10.3390/laboratories3010005)
Supplement: Supplementary files [file NIHMS2159182-supplement-Supplementary_files.zip › Table_S2.pdf]

Table S2. Sensitivity, specificity, and percent agreement of OLA-Simple (OS) compared to Sanger Sequencing across drug resistance mutations. Test performance indicating the sensitivity, specificity and percent agreement of OLA-Simple test results compared to Sanger sequencing (excluding the tie-breaker, sensitive ELISA-based OLA) across the 7 HIV-DR mutations for the samples tested.

	Mutation detected by OS / Sanger sequencing		Wild-type codon by OS / Sanger sequencing		Percent Agreement (95% CI)
Resistance Mutation	n/N Positive by OS (TP)	Sensitivity (95% CI)	n/N Negative by OS (TN)	Specificity (95% CI)	
K65R	6/6	100% (54.1, 100)	114/116	98.3% (94.0, 99.8)	98.4% (94.2, 99.8)
L74VI	25/25	100% (86.3, 100)	101/101	100% (96.4, 100)	100% (97.1, 100)
Y115F	11/12	91.7% (61.5, 99.8)	114/114	100% (96.8, 100)	99.2% (95.6, 99.9)
K103N	65/67	97.0% (89.6, 99.6)	51/63	81.0% (69.1, 89.7)	89.2% (82.6, 94.0)
Y181C	21/22	95.5% (77.1, 99.9)	104/106	98.1% (93.3, 99.8)	97.7% (93.3, 99.5)
M184V	87/89	97.8% (89.1, 98.8)	36/39	92.3% (79.1, 98.4)	96.1% (89.2, 97.8)
G190A	33/34	91.7% (84.7, 99.9)	89/93	95.8% (89.3, 98.8)	94.7% (91.0, 98.7)
**Total n/N**	**248/255**	**97.3% (94.4, 98.9)**	**609/632**	**96.4% (94.5, 97.7)**	**96.4% (95.0, 97.5)**
n/N = OLA-simple/Sanger sequencing; TP: True Positive; TN: True Negative; Clopper-Pearson Confidence Interval (CI) was used					
