# Supplementary material for: Oligonucleotide Ligation Assay (OLA)-Simple: Field Implementation, Usability, and Performance of a near Point-of-Care HIV Drug Resistance Assay in Kenya
Source: Laboratories. Author manuscript; Available in PMC 2026 Apr 3. (PMC13046437; doi:10.3390/laboratories3010005)
Supplement: Supplementary files [file NIHMS2159182-supplement-Supplementary_files.zip › Table_S3.pdf]

Table S3. Positive predictive value (PPV) and negative predictive value (NPV) of OLA-Simple (OS) compared to Sanger Sequencing across drug resistance mutations. Test performance indicating the positive and negative predictive value of OLA-Simple testing compared to Sanger sequencing (excluding the tie-breaker, sensitive ELISA-based OLA) across the 7 HIV-DR mutations for the samples tested.

Resistance Mutation	CS positives		CS negatives	
	n/N positive by OS (TP) and negative by CS (FP)	PPV	n/N positive by CS (TN) & negative by OS (FN)	NPV
K65R	6/8	75.0%	114/114	100%
L74VI	25/25	100%	101/101	100%
Y115F	11/11	100%	114/115	99.1%
K103N	65/77	84.4%	51/53	96.2%
Y181C	21/23	91.3%	104/105	99.0%
M184V	87/90	96.7%	36/38	94.7%
G190A	33/37	89.2%	89/90	98.9%

TP: True Positive; FP: False Positive; TN: True Negative; FN: False Negative; PPV: Positive Predictive Value; NPV: Negative Predictive Value; CS: Consensus sequencing or Sanger Sequencing
