# Supplementary material for: Oligonucleotide Ligation Assay (OLA)-Simple: Field Implementation, Usability, and Performance of a near Point-of-Care HIV Drug Resistance Assay in Kenya
Source: Laboratories. Author manuscript; Available in PMC 2026 Apr 3. (PMC13046437; doi:10.3390/laboratories3010005)
Supplement: Supplementary files [file NIHMS2159182-supplement-Supplementary_files.zip › Figure_S1.pdf]

A 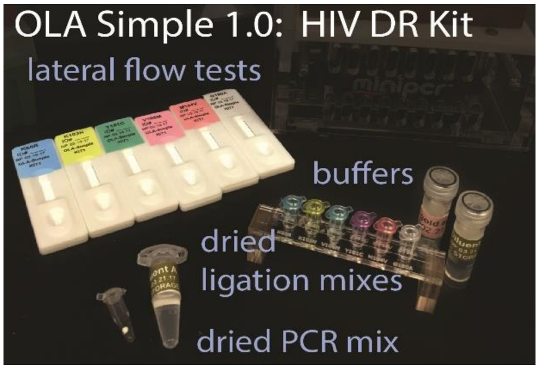

B 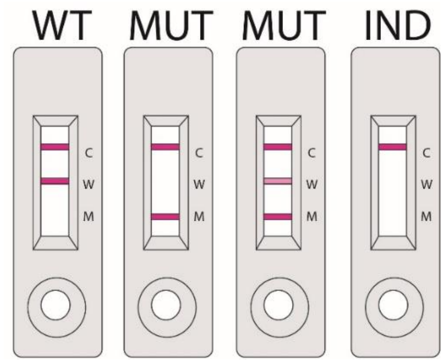

Figure S1. Picture of OLA-Simple test detection kit content and a diagrammatic representation of the possible band combinations on detection strips. (A): The OLA-Simple test kit uses lyophilized PCR reagents for RNA amplification, dried OLA probes for each mutation, and lateral flow cartridges for visual detection of each mutation. Liquid reagents include buffer for the ligation reaction and gold nanoparticles for lateral flow strip detection. **(B)**: Each lateral flow strip contains three detection lines: a control line (C) that binds the gold nanoparticles, a wild-type line (W) that binds wild-type ligated probes, and a mutant line (M) that binds mutant ligated probes. If the W line is present but the M line is absent, the sample is classified as wild-type (WT). If the M line is present to any degree, the sample is classified as mutant (MUT) regardless of the status of the W line. If neither the W nor the M line is present, the sample is classified as indeterminate (IND) due to failure at some point in the test.
