# Supplementary material for: Oligonucleotide Ligation Assay (OLA)-Simple: Field Implementation, Usability, and Performance of a near Point-of-Care HIV Drug Resistance Assay in Kenya
Source: Laboratories. Author manuscript; Available in PMC 2026 Apr 3. (PMC13046437; doi:10.3390/laboratories3010005)
Supplement: Supplementary files [file NIHMS2159182-supplement-Supplementary_files.zip › Table_S1.pdf]

Table S1. Nielsen's usability framework domains and example questions asked in our OLA-Simple usability survey.

Domain	Objective	Example question for OLA-Simple usability survey
Learnability	The system is easy to learn so users can start using it quickly.	How long did it take to run OLA-Simple for the first time?
Efficiency	Once the user has learned to use the system, there should be a high level of productivity.	After learning how to run OLA-Simple, how long does it take to run it once?
Memorability	The system should be easy to remember so users will still know how to use it even after a break period.	After a period of absence, would it be easy to run OLA-Simple again?
Error	The system should have a low error rate and any errors made should be easy to recover from and not catastrophic.	Can you see any areas where errors could easily be made that would require you to start over?
Satisfaction	Users should like using the system.	How was your overall experience running OLA-Simple?

Note: To evaluate usability of the OLA-Simple, anonymous survey questionnaires were designed according to Nielsen's usability framework, which entailed domains of learnability, efficiency, memorability, error, and satisfaction. Each domain is outlined, including their definitions and example questions that were used in the survey – corresponding to the domain.
