# Supplementary material for: Oligonucleotide Ligation Assay (OLA)-Simple: Field Implementation, Usability, and Performance of a near Point-of-Care HIV Drug Resistance Assay in Kenya
Source: Laboratories. Author manuscript; Available in PMC 2026 Apr 3. (PMC13046437; doi:10.3390/laboratories3010005)
Supplement: Supplementary files [file NIHMS2159182-supplement-Supplementary_files.zip › OLA-Simple study participation Consent Forms.pdf]

Written Consent Form, OLA Simple Survey

Study Title: Optimizing viral load suppression in Kenyan children on antiretroviral therapy (Opt4Kids)-OLA Simple

Investigators	Institution Affiliation	Contact	Role
Rena Patel, MD, MPH	University of Washington<br>Seattle, USA	rcpatel@uw.edu<br>+1 650 353 0718	Principal-Investigator
Lisa Abuogi, MD, MSc	University of Colorado, Denver<br>Aurora, USA	lisa.abuogi@ucdenver.edu	Principal-Investigator
Patrick Oyaro, MBChB, MPH	LVCT Health<br>Mombasa, Kenya	patrickoyaro@gmail.com	Site Principal-Investigator

PART I: INFORMATION SHEET

Researchers' statement

We are asking you to participate in a research study to evaluate the performance of a point-of-care assay, called OLA Simple, to test for HIV drug resistance mutations. We will also be asking you about your thoughts on the OLA Simple training and how to improve the assay, its software, and training for the future via a survey. This form provides you with information about the study. Please read the information below and ask questions about anything you don't understand before deciding whether or not to take part.

Why is this study being done?

We are interested in developing and implementing a point-of-care HIV drug resistance assay to improve access to HIV drug resistance testing in settings like Kenya.

How many people will take part in the study?

Up to approximately 20 people will participate in the study.

What happens if you agree to participate?

If you accept to participate in the study, you will undergo training on the use of OLA Simple, participate in conducting testing on OLA Simple, including for quality control, and complete a survey to assess your experiences with the training and how to improve the assay, its software, and the training for the future.

What are the possible discomforts or risks?

Discussing some of the aspects of training may be uncomfortable for you.

What are the possible benefits of the study?

This study will help the researchers gain a better understanding of the accuracy of the assay in detecting HIV drug resistance mutations and how to improve the assay and training for future use. The assay results will not be shared with the participants or their providers, and not influence any clinical decision-making.

Will I be paid for being in the study?

No, you will not be compensated for your participation in the study, but you will be compensated for transport for training and testing.

Will I have to pay for anything?

No, taking part in the study will not cost you anything.

Is my participation voluntary?

Yes, taking part in this study is voluntary. You have the right to choose not to take part in this study. If you choose to take part, you have the right to stop at any time. If you refuse or decide to withdraw later, you will not lose any benefits or rights to which you are entitled.

Who will see my research information?

We would include the results in our study reporting, including at meetings, conferences, and for publication(s), and it might be shared with researchers in the future, but we will not mention your name or any identifiable

information. No identifiable information will be collected from you, so no one will be able to track the responses to you. Your information will be kept private when any information is presented.

Certificate of Confidentiality

We have a Certificate of Confidentiality from the United States NIH. These protections only apply to data held in the United States.

This helps us protect your privacy. The Certificate means that we do not have to give out identifying information about you even if we are asked to by a court of law in the United States. We will use the Certificate to resist any demands for identifying information.

We can't use the Certificate to withhold your research information if you give your written consent to give it to an insurer, employer, or other person. Also, you or a member of your family can share information about yourself or your part in this research if you wish.

There are some limits to this protection. We will voluntarily provide the information to:

- A member of the United States government who needs it in order to audit or evaluate the research;
- Individuals at the universities, the funding agency, and other groups involved in the research, if they need the information to make sure the research is being done correctly; and
- To relevant authorities as required by other Federal, State, or local laws.

Who do I call if I have questions?

You may ask any questions you have now. If you have questions, concerns, or complaints later, you may call the study coordinator at the telephone number (057) 2021036.

If you have questions about your rights as a research participant or if you wish to voice your concerns about the study, please contact the office of the Research Officer at AMREF Kenya at (020) 699 4000 or write to P.O Box 30125-00100, Nairobi, Kenya. You can also contact the UW Human Subjects Division at +1206 543 0098, write to P.O. Box 359470, Seattle, WA, USA 98195, or email hsdinfo@uw.edu.

PART II: CERTIFICATE OF CONSENT

Agreement to be in this study

I have read this paper about the study or it was read to me. I understand the possible risks and benefits of this study. I know that my participation in this study is voluntary. I choose to take part in this study. I will get a copy of this consent form.

Participant Name:________________________ Date:____________

Participant Signature:____

Consenting Staff Name:________________________ Date:_________________

Staff Signature:____

Written Consent Form, OLA Simple Survey

Study Title: Optimizing viral suppression for pregnant and postpartum women living with HIV through point-of-care viral load testing (Opt4Mamas)-OLA Simple

Investigators	Institution Affiliation	Contact	Role
Rena Patel, MD, MPH	University of Washington<br>Seattle, USA	rcpatel@uw.edu<br>+1 650 353 0718	Principal-Investigator
Lisa Abuogi, MD, MSc	University of Colorado, Denver<br>Aurora, USA	lisa.abuogi@ucdenver.edu	Principal-Investigator
Patrick Oyaro, MBChB, MPH	LVCT Health<br>Mombasa, Kenya	patrickoyaro@gmail.com	Site Principal-Investigator

PART I: INFORMATION SHEET
<u>Researchers' statement</u>
We are asking you to participate in a research study to evaluate the performance of a point-of-care assay, called OLA Simple, to test for HIV drug resistance mutations. We will also be asking you about your thoughts on the OLA Simple training and how to improve the assay, its software, and training for the future via a survey. This form provides you with information about the study. Please read the information below and ask questions about anything you don't understand before deciding whether or not to take part.

Why is this study being done?
We are interested in developing and implementing a point-of-care HIV drug resistance assay to improve access to HIV drug resistance testing in settings like Kenya.

How many people will take part in the study?
Up to approximately 20 people will participate in the study.

What happens if you agree to participate?
If you accept to participate in the study, you will undergo training on the use of OLA Simple, participate in conducting testing on OLA Simple, including for quality control, and complete a survey to assess your experiences with the training and how to improve the assay, its software, and the training for the future.

What are the possible discomforts or risks?
Discussing some of the aspects of training may be uncomfortable for you.

What are the possible benefits of the study?
This study will help the researchers gain a better understanding of the accuracy of the assay in detecting HIV drug resistance mutations and how to improve the assay and training for future use. The assay results will not be shared with the participants or their providers, and not influence any clinical decision-making.

Will I be paid for being in the study?
No, you will not be compensated for your participation in the study, but you will be compensated for transport for training and testing.

Will I have to pay for anything?
No, taking part in the study will not cost you anything.

Is my participation voluntary?
Yes, taking part in this study is voluntary. You have the right to choose not to take part in this study. If you choose to take part, you have the right to stop at any time. If you refuse or decide to withdraw later, you will not lose any benefits or rights to which you are entitled.

Who will see my research information?
We would include the results in our study reporting, including at meetings, conferences, and for publication(s), and it might be shared with researchers in the future, but we will not mention your name or any identifiable

information. No identifiable information will be collected from you, so no one will be able to track the responses to you. Your information will be kept private when any information is presented.

Certificate of Confidentiality
We have a Certificate of Confidentiality from the United States NIH. These protections only apply to data held in the United States.

This helps us protect your privacy. The Certificate means that we do not have to give out identifying information about you even if we are asked to by a court of law in the United States. We will use the Certificate to resist any demands for identifying information.

We can't use the Certificate to withhold your research information if you give your written consent to give it to an insurer, employer, or other person. Also, you or a member of your family can share information about yourself or your part in this research if you wish.

There are some limits to this protection. We will voluntarily provide the information to:

- A member of the United States government who needs it in order to audit or evaluate the research;
- Individuals at the universities, the funding agency, and other groups involved in the research, if they need the information to make sure the research is being done correctly; and
- To relevant authorities as required by other Federal, State, or local laws.

Who do I call if I have questions?
You may ask any questions you have now. If you have questions, concerns, or complaints later, you may call the study coordinator at the telephone number (057) 2021036.

If you have questions about your rights as a research participant or if you wish to voice your concerns about the study, please contact the office of the Research Officer at AMREF Kenya at (020) 699 4000 or write to P.O Box 30125-00100, Nairobi, Kenya. You can also contact the UW Human Subjects Division at +1206 543 0098, write to P.O. Box 359470, Seattle, WA, USA 98195, or email hsdinfo@uw.edu.

PART II: CERTIFICATE OF CONSENT

Agreement to be in this study

I have read this paper about the study or it was read to me. I understand the possible risks and benefits of this study. I know that my participation in this study is voluntary. I choose to take part in this study. I will get a copy of this consent form.

Participant Name:________________________ Date:____________

Participant Signature:____

Consenting Staff Name:________________________ Date:_________________

Staff Signature:____

Consent Form (English)

Study Title: Optimizing viral load suppression in Kenyan children on antiretroviral therapy (Opt4Kids)

Investigators	Institution Affiliation	Contact	Role
Rena Patel, MD, MPH	University of Washington Seattle, USA	rcpatel@uw.edu +1 650 353 0718	Principal-Investigator
Lisa Abuogi, MD, MSc	University of Colorado, Denver Aurora, USA	Lisa.abuogi@ucdenver.edu	Principal-Investigator
Irene Mukui, MBChB	National AIDS and STI Control Programme Nairobi, Kenya	imukui@nascop.or.ke	Co-Investigator
Patrick Oyaro, MBChB	RCTP-FACES NGO Kisumu, Kenya	patrickoyaro@gmail.com	Co-Investigator

---

We are asking for your child to be in a research study. This form provides you with information about the study. A member of the research team will describe this study to you and answer all of your questions. Please read the information below and ask questions about anything you don't understand before deciding whether or not to all ow your child to take part.

Why is this study being done?

This study plans to learn more about viral load testing and drug resistance for children 1-14 years of age on antiretroviral medications for HIV. This research is being done by investigators at the University of Washington, the University of Colorado, and in Kenya. You are being asked to be in this research study because your child is 1-14 years old, living with HIV and on medications for HIV.

A description of this clinical trial will be available on http://www.clinicaltrials.gov, as required by U.S. Law. This Web site will not include information that can identify you. At most, the Web site will include a summary of the results. You can search this Web site at any time

How many children will take part in the study?

Approximately 700 children from your area will participate in the study.

What happens if my child joins this study?

If you join the study, your child will be randomly placed in one of two study options. The first option is regular care and the second option is HIV care with point-of-care viral load testing.

If your child is placed in regular care group:

- Your child will continue to get HIV care at this clinic as you always do
- Your child will get viral load and other lab tests when the clinician or nurse thinks necessary and orders them
- Your child may have a blood sample taken for viral load test or drug resistance testing once or twice during the study period
- The study team will collect your information from the medical chart
- Someone from the study will ask you and your child some extra questions at regular visits (which will take about 30 minutes)

If your child is placed in point-of-care viral load group:

- Your child will continue to get HIV care at this clinic as you always do

- Your child will have a blood sample (of approximately 1-2 teaspoons) taken for viral load tests about every 3 months at regular clinic visits
- If your child's viral load is high,
  - You and your clinic provider will be notified, and you will discuss ways to lower your child's viral load
  - Your child's blood may be sent to check for drug resistance
- After testing for this study is completed, any remaining samples may be stored for future studies
- The study team will collect your child's information from the medical chart
- Someone from the study will ask you and your child some extra questions at your regular clinic visits (which will take about 30 minutes)

If your child is in this study, you will be asked questions about:

- Your family, your home, and your child's school
- Your child's HIV medications and how you give them
- Challenges with taking HIV medications

How long will the study last?

The study will last approximately one year.

What are the possible discomforts or risks?

Discomforts your child may experience while in this study include:

- Taking a blood sample for viral load tests will hurt a little bit. It is the same as when your child gets blood samples taken for other tests for HIV care. If your child is in the point-of-care viral load group, he/she might get this done a little bit more often than regular care.

- You or your child might face some social risks including disruption of family, discrimination, and/or physical harm involved, if your child's HIV status, or other personal details were to be disclosed outside of the research setting.

What are the possible benefits of the study?

This study is designed for the researcher to learn more about using point-of-care viral load testing and drug resistance testing in children on HIV medications. This study is not designed to treat any illness or to improve your health. Also, there may be risks, as discussed above.

Who is paying for this study?

This research is being funded by the United States National Institutes of Health (NIH).

Will your child be paid for being in the study?

Your child will not be paid to be in the study.

Will your child have to pay for anything?

It will not cost you or your child anything to be in the study.

Is your child's participation voluntary?

Taking part in this study is voluntary. You have the right to choose not to have your child take part in this study. If you choose to have your child take part, you have the right to stop at any time. If you refuse or decide to withdraw later, you will not lose any benefits or rights to which you or your child are entitled.

If you leave this study, you and your child will still receive your normal medical care. The only medical care that your child will lose is the additional medical care your child is getting as part of this study.

Can I be removed from this study?

The study team may decide to stop your child's participation without your permission if the study team thinks that being in the study may cause your child harm, or for any other reason. A study team member will call you in such a case.

Who do I call if I have questions?

You may ask any questions you have now. If you have questions, concerns, or complaints later, you may call the study coordinator at the telephone number (057) 2021036.

If you have questions about your rights as a research participant or if you wish to voice your concerns about the study, please contact the office of the Research Officer at AMREF Kenya at (020) 699 4000 or write to P.O Box 30125-00100, Nairobi, Kenya. You can also contact the UW Human Subjects Division at +1206 543 0098, write to P.O. Box 359470, Seattle, WA, USA 98195, or email hsdinfo@uw.edu.

Who will see my research information?

We will do everything we can to keep your child's records a secret, but it cannot be guaranteed. The results from the research may be shared at a meeting or published in articles. Your child's name will be kept private when information is presented.

The data we collect will be used for this study but may also be important for future research. Your data may be used for future research or distributed to other researchers for future study without additional consent if information that identifies you is removed from the data.

CERTIFICATE OF CONFIDENTIALITY

We have a Certificate of Confidentiality from the United States NIH. These protections only apply to data held in the United States. This helps us protect your privacy. The Certificate means that we do not have to give out identifying information about you even if we are asked to by a court of law in the United States. We will use the Certificate to resist any demands for identifying information.

We can't use the Certificate to withhold your research information if you give your written consent to give it to an insurer, employer, or other person. Also, you or a member of your family can share information about yourself or your part in this research if you wish.

There are some limits to this protection. We will voluntarily provide the information to:

- A member of the United States government who needs it in order to audit or evaluate the research;

- Individuals at the universities, the funding agency, and other groups involved in the research, if they need the information to make sure the research is being done correctly; and
- To relevant authorities as required by other Federal, State, or local laws.

Agreement to be in this study

I have read this paper about the study or it was read to me. I understand the possible risks and benefits of this study. I know that my child's participation in this study is voluntary. I choose to allow my child to be in this study. I will get a copy of this consent form.

Child's Name: ____________________________

Parent/Caregiver Name:________________________ Date:____________

Parent/Caregiver Signature:____

Consenting Staff Name:________________________ Date:_________________

Staff Signature:____

Specimen storage, shipment, and use of your data and samples for future studies (study staff to mark for participant if consenting orally)

Please initial one option:
____________ I **DO** agree to store, ship and test my samples overseas.

____________ I **DO NOT** agree to store, ship and test my samples overseas.

Please initial one option:
____________ I **DO** agree to the use my data and samples for future research.

____________ I **DO NOT** agree to the use my data and samples for future research.

If consented orally

I have witnessed the accurate reading of the consent form to the participant and the individual has had the opportunity to ask questions. I confirm that the individual has given consent freely.

Name of witness: _____________________________

Signature of witness: __________________________

Date: _______________________________________

Thumbprint of participant

Consent Form (English)

Consenting Staff Name:________________________ Date:_________________

Staff Signature:____

Assent Form (English)

Assent Form

(Children 13-14 years)

Study Title: Opt4Kids

What is this study about?
A study is when doctors collect a lot of information to learn more about something. You are being asked if you want to be in this study. The goal of this study is to find out if doing more frequent and faster tests will improve the health of children and adolescents.

Why are you asking me?
You are being asked to be in the study because you are getting care at this clinic.

What do I have to do or what will happen to me?
If you are in the study, you will:

- *Be put in the regular care or the study group*
- *You will be in the study for about a year*

If you are in the regular care group:

- *You will continue to get care at this clinic as you always do*
- *You will get lab tests when the clinician or nurse orders them*
- *You may have a blood sample taken for tests once or twice during the study period*
- *The study team will collect your information from the medical chart*
- *Someone from the study will ask you and your parent or caregiver some extra questions (which will take about 30 minutes)*

If you are in the study group:

- *You will continue to get care at this clinic as you always do*
- *You will have a blood sample (of approximately 1-2 teaspoons) taken for tests about every 3 months*
- *Any blood remaining after completing the tests may be saved for future studies*
- *The study doctors will collect your information from the medical chart*
- *Someone from the study will ask you and your parent or caregiver some extra questions (which will take about 30 minutes)*

If you are in this study, you will be asked questions about:

- Your family, your home, and your school
- Your medications and how you take them
- Challenges with taking any medications

You don't have to answer any questions that you don't want to, and you can stop at any time.

Will this hurt?
Taking a blood sample for tests will hurt a little bit. It is the same as when you get blood samples taken for other tests for your care. If you are in the study group, you might get this done a little bit more often than regular care.

Can I ask questions?
You can ask any questions that you have now about the study.

If you have a question later, you can ask and get an answer. If you want to, you can call the study coordinator at the telephone number (057) 2021036.

Do I have to do this?

Assent Form (English)

You do not have to be in this study. No one will be mad at you if you say no. You can choose to stop at any time. Just tell the researcher if you want to stop.

CERTIFICATE OF CONFIDENTIALITY

We have a Certificate of Confidentiality from the United States government. This helps us protect your privacy. In some cases, we may voluntarily provide the information to:

- People in government or those involved in the research who make sure the research is being done correctly; and
- To other people as required by law.

Assent Form (English)

Do you want to be in the study at this time? (Check one box)

☐ Yes ☐ No

You will get a copy of this form to keep.

Child's Name:___________________________ Date:____________

Child's Signature:____

Specimen storage, shipment, and use of your data and samples for future studies (study staff to mark for participant if consenting orally)

Please initial one option:

____________ I **DO** agree to store, ship and test my samples overseas.

____________ I **DO NOT** agree to store, ship and test my samples overseas.

Please initial one option:

____________ I **DO** agree to the use my data and samples for future research.

____________ I **DO NOT** agree to the use my data and samples for future research.

I have explained the research at a level that is understandable by the child and believe that the child understands what is expected during this study.

Consenting Staff Name:_________________________ Date:__________________

Staff Signature:_____________________________

If assented orally

I have witnessed the accurate reading of the consent form to the participant and the individual has had the opportunity to ask questions. I confirm that the individual has given consent freely.

Name of witness: ______________________________

Signature of witness: ___________________________

Date: ____

Thumbprint of participant

Consenting Staff Name:_________________________ Date:__________________

Staff Signature:____

Consent Form, Study Participation (English)

Study Title: Optimizing viral suppression for pregnant and postpartum women living with HIV through point-of-care viral load testing (Opt4Mamas)

Investigators	Institution Affiliation	Contact	Role
Rena Patel, MD, MPH	University of Washington Seattle, USA	rcpatel@uw.edu +1 650 353 0718	Principal-Investigator
Lisa Abuogi, MD, MSc	University of Colorado, Denver Aurora, USA		Co-Investigator
Patrick Oyaro, MBChB, MPH	RCTP-FACES NGO Kisumu, Kenya		Co-Investigator

PART I: INFORMATION SHEET

Researchers' statement

We are asking you to participate in a research study. This form provides you with information about the study. A member of the research team will describe this study to you and answer all of your questions. Please read the information below and ask questions about anything you don't understand before deciding whether or not to take part.

Why is this study being done?

This study plans to learn more about viral load and drug resistance mutation testing for pregnant and postpartum women on antiretroviral medications for HIV. This research is being done by investigators at the University of Washington, the University of Colorado, and in Kenya. You are being asked to be in this research study because you are living with HIV, on medications for HIV, and currently pregnant.

How many women will take part in the study?

Approximately 700 women will participate in the study.

What happens if I join this study?

If you join the study, you will join one of two study groups depending on when you enroll in the study. The first group is regular care and will enroll women in the first half of the study. The second group is regular care with point-of-care viral load testing and will enroll women in the second half of the study.

If you are in regular care group:

- You will continue to get HIV care at this clinic as you always do
- You will get viral load and other lab tests when the clinician or nurse thinks necessary and orders them
- You may have a blood sample taken for viral load test 2-3 times during the study period
- After testing for this study is completed, any remaining samples may be stored for future studies
- The study team will collect information about you and your infant from the medical chart
- Someone from the study will ask you some extra questions(detailed below) at regular visits (which will take about 30 minutes)

If you are in the point-of-care viral load group:

- You will continue to get HIV care at this clinic as you always do
- You will have a blood sample (of approximately 1-2 tablespoons) taken for viral load and drug resistance tests about every 1-3 months at regular clinic visits
- If your viral load is high or you have drug resistance, you and your clinic provider will be notified, and you will discuss ways to lower your viral load

- After testing for this study is completed, any remaining samples may be stored for future studies
- The study team will collect information about you and your infant from the medical chart
- Someone from the study will ask you some extra questions at your regular clinic visits (which will take about 30 minutes)

If you take part in this study, regardless of which group you join, you will be asked questions about:

- Your background, medical history, and current pregnancy
- Your HIV medications and how you take them
- Challenges with taking HIV medications

We would like to follow the information recorded about your infant in the medical chart, but no tests or blood samples will be taken from your infant.

How long will the study last?
The study will last approximately one year.

What are the possible discomforts or risks?
You may experience some discomfort while having your blood sample taken. It is the same as when you get blood samples taken for other tests for HIV care. If you are in the point-of-care viral load group, you might get this done more often than regular care. You may have a bruise where the needle goes into your arm. On rare occasions, infection may occur at the site of blood draw. All efforts will be made to ensure that this does not happen by using standard procedures for drawing blood. In the unlikely event that the infection occurs where the blood was taken, study staff will help you get the necessary treatment at the appropriate health facility.
You may become embarrassed, worried or anxious when talking about certain topics during the interview. You do not have to answer any questions that make you uncomfortable or that you do not want to answer. You may leave the interview at any time for any reason. Interviews will take place in a private room. We will make every effort to protect your confidentiality. However, it is possible that others may learn of your participation in this study and think that you have HIV. It is therefore possible, that others may treat you unfairly or discriminate against you. However, the risk for this happening is very small.

What are the possible benefits of the study?
This study is designed for the researcher to learn more about using point-of-care viral load and drug resistance testing for women on HIV medications. This study is not designed to treat any illness or to improve your health. Also, there may be risks, as discussed above.

Who is paying for this study?
This research is being funded by the United States National Institutes of Health (NIH).

Will I be paid for being in the study?
No, you will not be paid to be in the study.

Will I have to pay for anything?
No, taking part in the study will not cost you anything.

Is my participation voluntary?
Yes, taking part in this study is voluntary. You have the right to choose not to take part in this study. If you choose to take part, you have the right to stop at any time. If you refuse or decide to withdraw later, you will not lose any benefits or rights to which you are entitled. If you leave this study, you will still receive your usual medical care.

Can I be removed from this study?

The study team may decide to remove you from the study without your permission if the study team thinks that being in the study may cause you harm or for any other reason. A study team member will call you in such a case.

How will my samples be used in the future?

After testing for this study is completed, any remaining samples may be stored for future studies. We cannot predict yet how exactly these samples will be used in the future, but we can reassure you that your identify will be protected. Before your samples leave the facility, they will be assigned a code and your name will not be on them. Your name will be linked to the code only at this facility and the link between your name and the research data will be destroyed after the records retention period required by law. After that time, the link between your name and the code on your data and samples will be destroyed. You will consent to future use of your samples using check boxes at the end of the consent form. You do not have to consent to future use in order to participate in this study

Who will see my research information?

We will do everything we can to keep your records a secret, but we may share it with others if required by law. The results from the research may be shared at a meeting or published in articles. Your information will be kept private when any information is presented.

Certificate of Confidentiality

We have a Certificate of Confidentiality from the United States NIH. These protections only apply to data held in the United States.

This helps us protect your privacy. The Certificate means that we do not have to give out identifying information about you even if we are asked to by a court of law in the United States. We will use the Certificate to resist any demands for identifying information.

We can’t use the Certificate to withhold your research information if you give your written consent to give it to an insurer, employer, or other person. Also, you or a member of your family can share information about yourself or your part in this research if you wish.

There are some limits to this protection.   We will voluntarily provide the information to:

- A member of the United States government who needs it in order to audit or evaluate the research;
- Individuals at the universities, the funding agency, and other groups involved in the research, if they need the information to make sure the research is being done correctly; and
- To relevant authorities as required by other Federal, State, or local laws.

Who do I call if I have questions?

You may ask any questions you have now. If you have questions, concerns, or complaints later, or if you feel you have been harmed by participating, you may call the study coordinator at the telephone number (057) 2021036.

If you have questions about your rights as a research participant or if you wish to voice your concerns about the study, please contact the office of the Research Officer at AMREF Kenya at (020) 699 4000 or write to P.O Box 30125-00100, Nairobi, Kenya. You can also contact the UW Human Subjects Division at +1206 543 0098, write to P.O. Box 359470, Seattle, WA, USA 98195, or email hsdinfo@uw.edu.

PART II: CERTIFICATE OF CONSENT

Agreement to be in this study

I have read this paper about the study or it was read to me. I understand the possible risks and benefits of this study. I know that my participation in this study is voluntary. I choose to take part in this study. I will get a copy of this consent form.

Participant Name:________________________ Date:____________

Participant Signature:____

Consenting Staff Name:________________________ Date:_________________

Staff Signature:____

Infant Follow-up

Please initial one option:

___________ I **DO** agree to allow access to my infant's medical records and follow-up.

___________ I **DO NOT** agree to allow access to my infant's medical records and follow-up.

Specimen storage, shipment, and use of your data and samples for future studies (study staff to mark for participant if consenting orally)

Please initial one option:

____________ I **DO** agree to store, ship and test my samples overseas.

____________ I **DO NOT** agree to store, ship and test my samples overseas.

Please initial one option:

____________ I **DO** agree to the use my data and samples for future research.

____________ I **DO NOT** agree to the use my data and samples for future research.

If consented orally

I have witnessed the accurate reading of the consent form to the participant and the individual has had the opportunity to ask questions. I confirm that the individual has given consent freely.

Name of witness: ______________________________

Thumbprint of participant

Signature of witness: ___________________________

Date: ______________________________________

Consenting Staff Name:_________________________ Date:__________________

Staff Signature:____
